# Structure-Induced Selectivity of Hydroxylated Covalent Organic Framework Nanofibers for Advanced Sensing Applications: An Experimental and Density Functional Theory Study

**DOI:** 10.1021/acsami.5c03407

**Published:** 2025-04-26

**Authors:** Nagy L. Torad, Tzu-Ling Yang, Moustafa A. Darwish, Putikam Raghunath, Ahsanulhaq Qurashi, Lamiaa Reda Ahmed, Ming-Chang Lin, Yusuke Yamauchi, Brian Yuliarto, Watchareeya Kaveevivitchai, Mohammad Abu Haija, Ahmed F. M. EL-Mahdy

**Affiliations:** a Department of Chemistry, Khalifa University of Science and Technology, P.O. Box 127788, Abu Dhabi, United Arab Emirates; b Center for Catalysis and Separation (CeCaS), Khalifa University of Science and Technology, P.O. Box 127788, Abu Dhabi, United Arab Emirates; c Chemistry Department, Faculty of Science, Tanta University, Tanta 31527, Egypt; d Department of Materials and Optoelectronic Science, National Sun Yat-Sen University, Kaohsiung 80424, Taiwan; e Physics Department, Faculty of Science, Tanta University, Al-Geish St., Tanta 31527, Egypt; f Department of Applied Chemistry, National Yang Ming Chiao Tung University, Hsinchu 30010, Taiwan; g Institute of Medical Science and Technology, National Sun Yat-Sen University, Kaohsiung 804201, Taiwan; h Australian Institute for Bioengineering and Nanotechnology, The University of Queensland, Brisbane QLD 4072, Australia; i Department of Materials Process Engineering, Graduate School of Engineering, Nagoya University, Nagoya 464-8603, Japan; j Advanced Functional Materials Laboratory, Engineering Physics Department, Faculty of Industrial Technology, Institut Teknologi Bandung, Bandung 40132, Indonesia; k Research Center for Nanoscience and Nanotechnology (RCNN), Institut Teknologi Bandung, Bandung 40132, Indonesia; l Department of Chemical Engineering, Hierarchical Green-Energy Materials (Hi-GEM) Research Center, Academy of Innovative Semiconductor and Sustainable Manufacturing, National Cheng Kung University, Tainan City 70101, Taiwan

**Keywords:** hydroxyl-functionalized covalent organic frameworks (HO-COFs), nanofibers, ethylenediamine (EDA), quartz crystal microbalance (QCM), sensors

## Abstract

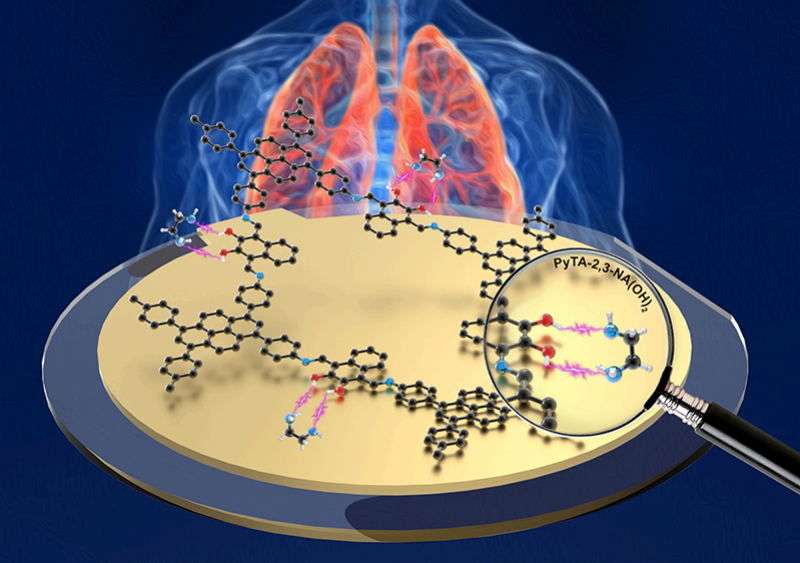

This study reports on the rational design of hydroxyl-functionalized covalent organic framework nanofibers (HO-COFs: PyTA-2,3-NA(OH)_2_ and PyTA-2,6-NA(OH)_2_) by a scalable solvothermal method. The resulting PyTA-2,3-NA(OH)_2_ HO-COF is more hydrophilic than the PyTA-2,6-NA(OH)_2_ HO-COF, which can effectively enhance the sensitivity of the sensor toward basic ethylenediamine (EDA). The fabricated HO-COF nanofiber-based quartz crystal microbalance sensor exhibits a rapid sensing response and a distinguished selectivity toward EDA vapor, arising from the strong hydrogen bonding interactions with the NH_2_ groups of EDA, as investigated by a wide variety of chemical analysis techniques and density functional theory calculations. The presence of exposed neighboring hydroxyl groups that face the same direction in the PyTA-2,3-NA(OH)_2_ HO-COF and the NH_2_ groups present in EDA exhibited efficient interactions. The PyTA-2,3-NA(OH)_2_ nanofiber with neighboring hydroxyl groups exhibits 1.6 times higher sensitivity to 100 ppm (ppm) EDA than PyTA-2,6-NA(OH)_2_ with hydroxyl groups in opposite directions, with a low limit of detection of 2.9 ppm. The PyTA-2,3-NA(OH)_2_ nanofiber structure has abundant active neighboring hydroxyl groups facing the same direction, making them favorable active sites for binding EDA molecules through strong hydrogen bond interactions. The color of the HO-COF changed after exposure to EDA vapor, as investigated by colorimetric assessment and naked-eye detection. These HO-COF nanofibers exhibit remarkable selectivity for EDA in the presence of other interfering chemical vapors and show high stability with only a 6.4% drop in sensitivity after 6 months. The adsorption of EDA on PyTA-2,3-NA(OH)_2_ nanofibers follows a pseudo-first-order kinetic model, with an adsorption rate about 8.0 times faster than PyTA-2,6-NA(OH)_2_ nanofibers. The findings of this study highlight the potential use of COFs, particularly those nanofibers with close neighboring hydroxyl groups, as effective sensing materials for the selective detection of harmful EDA.

## 1 Introduction

Recently reported microporous polymeric materials, such as covalent organic frameworks (COFs),^1−3^ covalent triazine frameworks (CTFs),^4,5^ intrinsic microporous organic polymers (PIMs),^6^ hyper cross-linked polymers (HCPs),^7,8^ and conjugated microporous polymers (CMPs),^9,10^ are promising materials with various applications as catalysis,^11^ energy storage/conversion,^12,13^ sensors,^14,15^ gas storage,^16^ and pollutant removal.^17^ In particular, COFs are crystalline organic polymers with systematically well-ordered pores and periodic skeletons having stable structures because of dynamic covalent chemistry.^18^ The remarkable thermal and chemical resistance, uniform micropores, large specific surface area, and extensive functionalization of COFs, particularly those with extended π-conjugated architectures, have attracted a lot of interest in gas adsorption, catalysis, energy storage and conversion, fluorescence, and biomedical and chemical sensors.^19,20^ Their diverse structures and building blocks enable a wide range of topologies that can be predesigned for multifaceted applications. Given their homogeneous nanoporous structure and high charge-carrier mobilities, COFs are widely studied for gas sensing/adsorption applications.^21,22^

Various studies have revealed significant advances in COFs due to their distinct morphologies and conformations. These facilitate the construction of confined places where elementary particles interact, developing novel molecular channels that impact their structure and function.^23−25^ The structural patterns within the COFs are governed by the varied topologies and dimensions of their knots and linkers. Two key aspects must be considered to achieve thermodynamic control during COF design and fabrication: the building unit structure and the synthesis technique, including reaction parameters.^26−28^ Characterized by stiffness and symmetrical multiconnectivities, the construction units of COF materials are pivotal in creating uniform pores. Several research groups have explored diverse synthetic strategies, such as solvothermal, ionothermal, and microwave approaches, since the COF synthesis through the solvothermal process by Yaghi et al. in 2005.^23^ They demonstrated the effective binding of minuscule building blocks using the concepts of dynamic covalent chemistry, resulting in extended porous crystalline COFs. However, unlike zero-dimensional (0-D) and one-dimensional (1-D) organic structures, higher-dimensional covalent polymers require *in situ* crystallization due to their insolubility and nonmelting character. A few years later, Furukawa and Yaghi synthesized two-dimensional (2-D) COFs from boronic-ester linkages formed by 2,3,6,7,10,11-hexahydroxytriphenylene and several di- and triboronic acid-functionalized building blocks.^29^ It has been reported that the crystalline triazine framework CTF-1 synthesized from 1,4-dicyanobenzene via the trimerization reaction of nitriles, was obtained by using an ionothermal synthesis technique.^5^ Subsequent advancements demonstrated the development of the 2-D imine-linked COF, which crystallizes in a hexagonal framework and is composed of 1,3,5-triformylbenzene and 1,4-diaminobenzene.^30^ Moreover, the reversible condensation of hydrazides with aldehydes to prepare hydrazones was also studied for the synthesis of 2-D COFs, yielding COF-42 and COF-43.^31^ Although they are a relatively small family, hydrazone COFs have found applications in sensing and catalysis.

Harmful gases, including H_2_S, NH_3_, NO_*x*_, SO_*x*_, CO_2_, CO, ethylenediamine (EDA), and chlorinated hydrocarbons, harm the environment and human health.^32,33^ EDA is a valuable raw material utilized in various industries as an intermediate to produce detergents, chelates, textile auxiliaries, agrochemicals, and polyamides.^34^ Despite the importance of EDA as a raw material, it is a highly flammable, corrosive chemical that can cause severe skin and eye damage. Inhaling EDA can lead to throat, nose, and lung irritation, resulting in coughing and shortness of breath.^35^ Therefore, sensitive and selective sensors are needed to detect EDA gas in various environments and industries. Thus, developing real-time and cost-effective chemical sensors for detecting EDA in an atmospheric environment with high sensitivity and selectivity is essential. Currently, most EDA detection methods rely on expensive and complicated instruments, including gas and liquid chromatography, mass spectrometry, fluorescence probes, and optical and gas-resistive sensors.^36−40^

The quartz crystal microbalance (QCM) sensor technology, known for its rapid response time, has gained the most attention among sensing methods due to its simplicity, low cost, high sensitivity, and ability to operate at room temperature.^14,41,42^ QCM sensor technology relies on measuring changes in the nanogram range of mass deposited on the surface of a piezoelectric quartz crystal by recording the change in QCM frequency corresponding to the mass of the adsorbed analyte. The QCM sensor technique demonstrated superior performance in various applications, such as enzyme detection, gas sensing, and polymerization reactions.^43^ In particular, QCM sensor have been extensively studied for real-time monitoring of deleterious chemicals and toxic gases, including mercury vapor, toluene vapor, ammonia gas, alcohols, amines, hydrogen cyanide, etc.^41,43−45^ QCM sensor are typically coated with nanoarchitectonics materials such as metal–organic frameworks (MOFs), MOF-derived carbons, COFs, CMPs, and carbon nanotubes due to their structural characteristics, such as a high surface area and pore volume per unit mass.^14,43,46−48^ Recently, COFs have been reported as sensitive coating materials for fabricating QCM sensor for the molecular recognition of individual chemical analytes.^49,50^ A new benzimidazole-containing COF has been reported for the QCM sensor for facile, rapid, highly sensitive, and selective detection of mustard gas, which typically poses a substantial concern for homeland security, and the detection of 2-chloroethyl ethyl sulfide (CEES), a typical hazardous mustard gas simulant, for the first time in 2019.^49^ The boronate-ester-linked 2-D COF thin films have been studied as active layers to fabricate QCM-based chemical sensor. Their porosity and Lewis acidity provide distinguished performance for the selective detection of volatile trimethylamine (TMA) at concentrations as low as 10 ppb, which can be explored and leveraged in a diverse range of thin-film devices.^51^ To the best of our knowledge, no study has yet demonstrated the effectiveness of highly nanoporous hydroxynaphthalene-based COFs as efficient gas sensors. Herein, we demonstrate their distinguished sensing activity toward hazardous EDA.

**Scheme 1:**
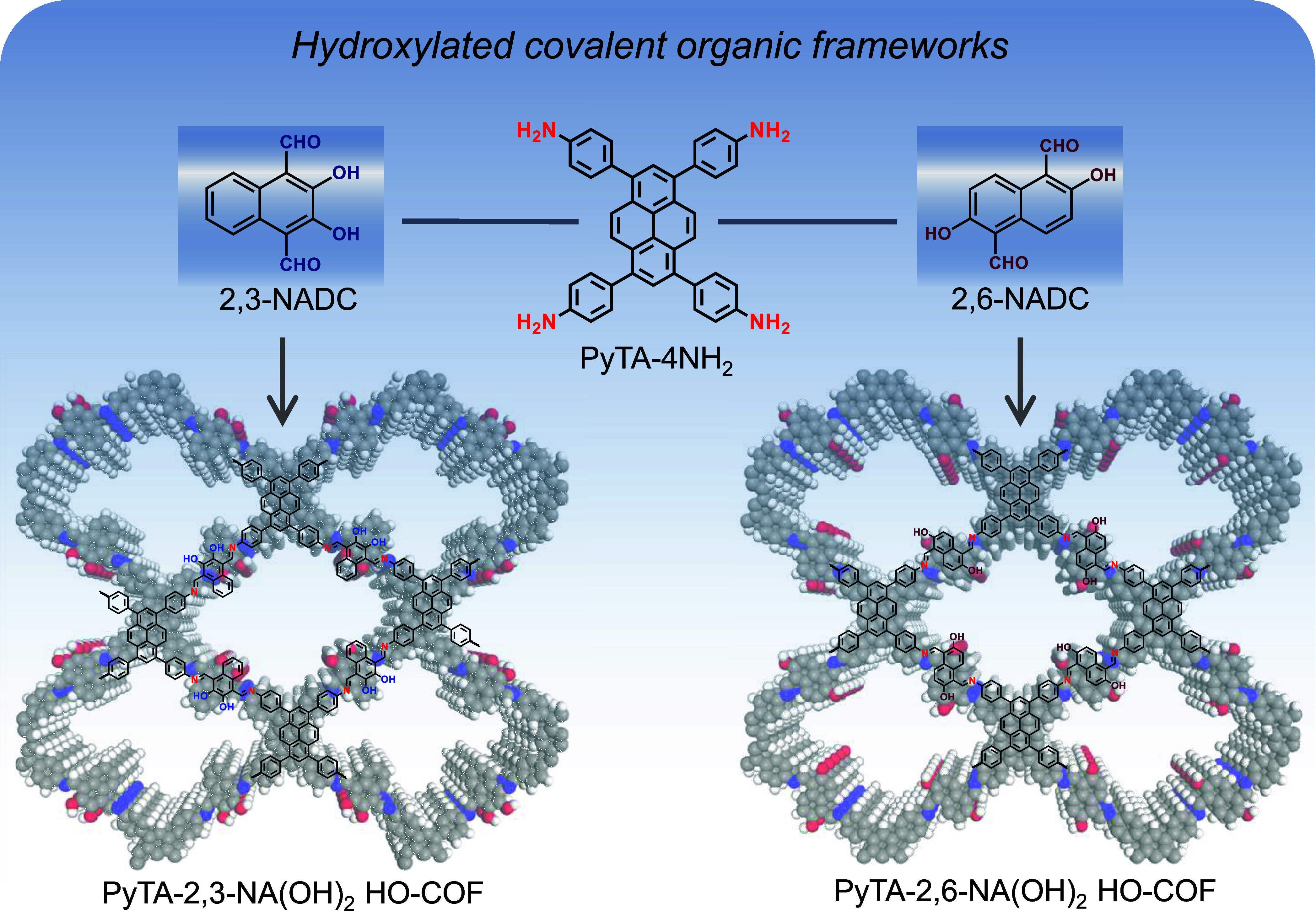
Schematic Representation of PyTA-2,3-NA(OH)_2_ (left) and PyTA-2,6-NA(OH)_2_ (right) HO-COF Structures

In this study, we report a scalable method to synthesize two novel pyrene-based HO-COF nanofibers, PyTA-2,3-NA(OH)_2_ and PyTA-2,6-NA(OH)_2_, through the solvothermal reaction of 2,3-dihydroxynaphthalene-1,4-dicarbaldehyde (2,3-NADC) or 2,6-dihydroxynaphthalene-1,5-dicarbaldehyde (2,6-NADC) with 1,3,6,8-tetrakis(4-aminophenyl)pyrene (PyTA-4NH_2_), respectively (Scheme 1). Both HO-COF materials exhibit high BET surface areas of up to 480 m^2^ g^–1^. We set out to design a structure-induced enhanced selectivity to EDA vapors by changing the directionality of hydroxyl groups to improve the sensing affinity and selectivity to EDA vapor via a strong hydrogen bonding interaction between EDA and hydroxyl groups inside the COFs. The pyrene unit was selected for our HO-COF-based selective sensor for EDA since it does not contain heteroatoms. COFs built up from heteroatom-containing monomers can dramatically reduce the detection selectivity of EDA if acidic chemical-vapor analytes have been present as interfering gases.^46^ The remarkable sensing activity, selectivity, and repeatability distinguish our fabricated PyTA-2,3-NA(OH)_2_-based QCM sensor due to considerable hydrogen bonding interactions of abundant active hydroxyl groups that have the same directionality on PyTA-2,3-NA(OH)_2_ nanofibers with both amino groups of EDA molecules. The sensitivity of the PyTA-2,3-NA(OH)_2_-based QCM sensor is the highest (2.57 Hz ppm^–1^) of the substances vapors studied, with a limit of detection (LoD) of EDA down to a subppm level of 2.9 ppm, with only a 6.4% drop in sensitivity after 6 months. The adsorption of EDA on PyTA-2,3-NA(OH)_2_ nanofibers follows a pseudo-first-order kinetic model, with an adsorption rate that is about 8.0 times faster than that of PyTA-2,6-NA(OH)_2_ nanofibers with low-abundant exposed hydroxyl groups. This work establishes an HO-COF nanofiber-based QCM sensor discrimination of EDA vapor using the QCM sensor technique.

## 2 Experimental Section

Comprehensive details of HO-COF materials, synthetic procedures, and characterizations are presented in the Supporting Information. Specifically, detailed synthetic methodologies for the preparation of 2,3-dihydroxynaphthalene-1,4-dicarbaldehyde (2,3-NADC), 2,6-dihydroxynaphthalene-1,5-dicarbaldehyde (2,6-NADC), and 1,3,6,8-tetrakis(4-aminophenyl)pyrene (PyTA-4NH_2_) as well as the synthesis of the PyTA-2,3-NA(OH)_2_ HO-COF and PyTA-2,6-NA(OH)_2_ COF are outlined in Sections “2. Synthetic Procedures” and “3. Characterization of HO-COFs”. Further details about the characterizations of the HO-COFs are presented in Section “4. Materials characterizations”. The experimental setup of the fabricated QCM-based gas sensor is demonstrated in Section “5. Quartz Crystal Microbalance Gas Sensor Experimental Setup”. Density functional theory (DFT) calculations are presented in Section “6. Computational Analysis Setup”.

## 3 Results and Discussion

### 3.1 Materials Characterization and Structural Assessment

**Figure 1:**
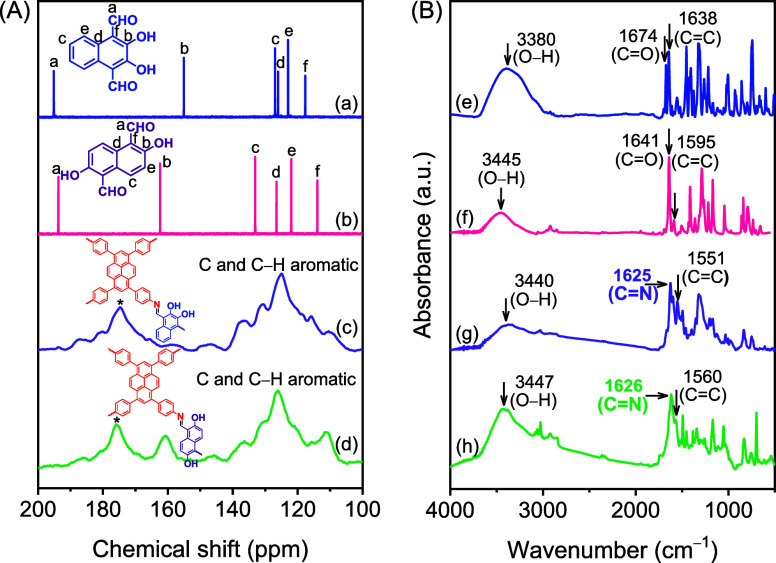
(A) Solid-state ^13^C NMR (a–d) and (B) FTIR spectra (e–h) of 2,3-NADC (blue line), 2,6-NADC (pink line), PyTA-2,3-NA(OH)_2_ (mauve line), and PyTA-2,6-NA(OH)_2_ (green line) HO-COFs.

In this study, the hydroxy naphthalene content significantly impacts the physical and chemical properties of our HO-COFs in terms of distinguished sensing activity and selectivity of individual deleterious chemical vapors. Therefore, the influence of abundant active hydroxyl group directionality in these COFs on the selective detection of hazardous vaporized EDA is investigated thoroughly. Two pyrene-based PyTA-2,3-NA(OH)_2_ and PyTA-2,6-NA(OH)_2_ HO-COF materials were synthesized quantitatively through the solvothermal reaction of the tetrakis(4-aminophenyl)pyrene linker (PyTA-4NH_2_) with two dihydroxynaphthalene dicarbaldehyde-based linkers, 2,3-NADC and 2,6-NADC, respectively, in a mixture of *n*-butanol and *o*-dichlorobenzene (1:1, *v*/*v*) as a cosolvent at 120 °C and in the presence of CH_3_COOH (6.0 M) as a catalyst (Scheme 1 and Schemes S1–S5 in the Supporting Information). The obtained pyrene-based HO-COFs were highly insoluble in common solvents, such as acetone, ethanol, methanol, tetrahydrofuran, and *N*,*N*′-dimethylformamide, demonstrating their strong cross-linking nature. The chemical compositions of the as-synthesized pyrene-based HO-COFs were analyzed by using solid-state ^13^C cross-polarization (CP)/magic-angle spinning (MAS), ^1^H NMR, and Fourier-transform infrared (FTIR) spectroscopies. The solid-state ^13^C NMR spectra reveal the complete polymerization of the monomer, demonstrating that carbon nucleus signals of the reactants are not detected reactants (Figure 1a–d and Figures S1–S8). The spectra of PyTA-2,3-NA(OH)_2_ and PyTA-2,6-NA(OH)_2_ HO-COFs show characteristic signals for the carbon nuclei of the aromatic C=N of the pyrene unit at 158.57 and 160.64 ppm, respectively (Figure 1c,d).^52^ The aryl carbon nuclei of aromatic C–H and C=C signals of the HO-COFs appear downfield at 125.92–130.58 ppm.^53^ Furthermore, the ^13^C NMR spectra of HO-COFs display two characteristic signals for their C–O units, at 147.18 and 146.24 ppm, respectively.

The FTIR spectra of HO-COFs indicate stretching vibrations of their aromatic C–OH, C=N, and C=C bonds at 3440 and 3447, 1625 and 1626, and 1551 and 1560 cm^–1^ for PyTA-2,3-NA(OH)_2_ and PyTA-2,6-NA(OH)_2_, respectively (Figure 1g,h and Figures S7 and S8).^53,54^ In addition, FTIR spectra show a complete coupling between 2,3-NADC, 2,6-NADC, and PyTA-4NH_2_, as evidenced by the disappearance of the characteristic absorption bands at 1674 and 1641 cm^–1^ of the aldehyde C=O bonds of 2,3-NADC and 2,6-NADC as well as the disappearance of C–N and N–H absorption bands at 1275 and 3343–3431 cm^–1^ of the starting PyTA-4NH_2_ monomer, respectively (Figures S7 and S8).

**Figure 2:**
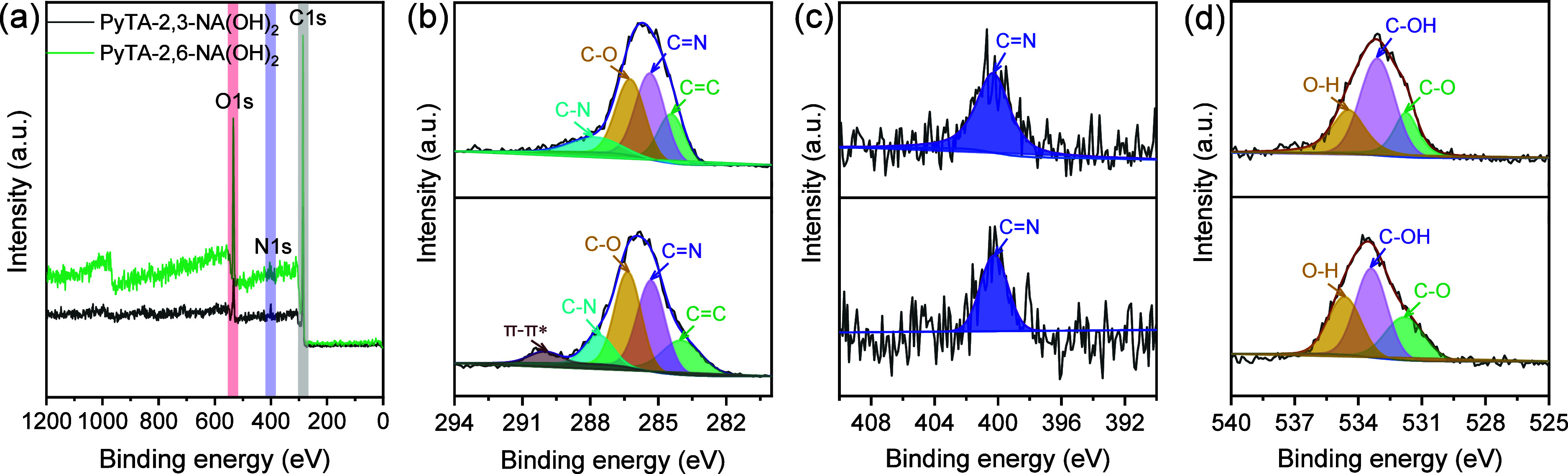
(a) Wide-scan XPS survey and high-resolution deconvoluted XPS spectra for (b) C 1s, (c) N 1s, and (d) O 1s of PyTA-2,3-NA(OH)_2_ (top) and PyTA-2,6 -NA(OH)_2_ HO-COF (bottom).

XPS measurements were conducted to understand the chemical composition and surface electronic states of HO-COFs, and the results are shown in Figure 2. The nitrogen of the HO-COF can create structural defects of unsaturated carbon atoms at edge sites. When exposed to air, these sites are very active in reacting with absorbed oxygen, forming oxygen-containing groups. The C 1s, N 1s, and O 1s states are observed with binding energies of 285.6, 400.4, and 533.7 eV in the wide-scan XPS survey spectra of PyTA-2,3-NA(OH)_2_ and PyTA-2,6-NA(OH)_2_ HO-COFs. Figure 2b–d shows the deconvoluted XPS peaks for C 1s, N 1s, and O 1s of both HO-COFs. The C 1s spectrum of the PyTA-2,3-NA(OH)_2_ HO-COF indicates the presence of several types of C species at 284.4, 285.3, 286.3, and 288.0 eV, assignable to C=C bonds, sp^2^ carbon-containing nitrogen atoms (C=N), C atoms attached to alcoholic oxygen (C–OH), and C–N bonds, respectively (Figure 2b (top)).^55^ The deconvoluted C 1s spectrum of PyTA-2,6-NA(OH)_2_ reveals four peaks that can be assigned to C=C, C=N, C–OH, and C–N species located at 284.0, 285.3, 286.3, and 287.7 eV, respectively.^56^ It has been noted that a satellite peak is observed at 290.1 eV as a result of the π–π* electronic transition (Figure 2b (bottom)). The deconvoluted N 1s spectra of both HO-COFs display a peak belonging to quaternary nitrogen, C=N.^57^ These peaks are located at 400.3 and 400.2 eV for PyTA-2,3-NA(OH)_2_ and PyTA-2,6-NA(OH)_2_, respectively (Figure 2c). Further, the O 1s spectrum of the PyTA-2,3-NA(OH)_2_ HO-COF (Figure 2d (top)) shows three peaks characteristic of C–O, C–OH, and O–H at 531.7, 533.1, and 534.4 eV, respectively, while the corresponding peaks for PyTA-2,6-NA(OH)_2_ (Figure 2d (bottom)) are situated at 531.8, 533.4, and 534.6 eV, respectively.

**Figure 3:**
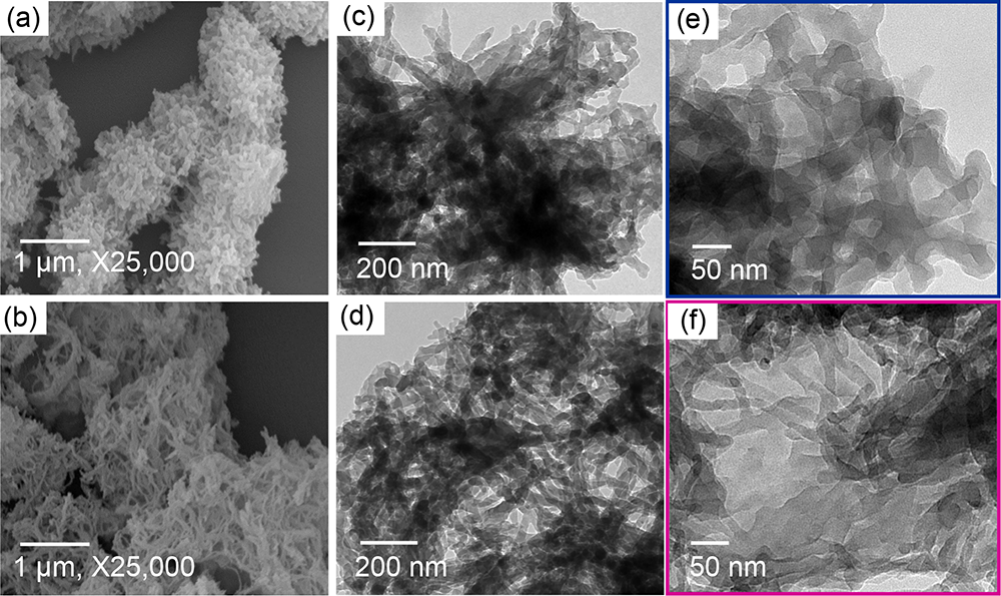
(a, b) SEM and (c–f) TEM images of PyTA-2,3-NA(OH)_2_ and PyTA-2,6-NA(OH)_2_ HO-COFs.

The morphologies of pyrene-based HO-COFs were studied by using field-emission scanning electron microscopy (FE-SEM) and transmission electron microscopy (TEM) (Figure 3). The FE-SEM images (Figure 3a,b) of PyTA-2,3-NA(OH)_2_ and PyTA-2,6-NA(OH)_2_ HO-COFs reveal the formation of nanostructured COFs with nanofiber morphologies of approximately 20 nm in width. TEM images of PyTA-2,3-NA(OH)_2_ show the growth of nanofibers with a diameter of about 19 nm (Figure 3c–e). The smooth surfaces and nanofiber morphologies are also confirmed for the PyTA-2,6-NA(OH)_2_ HO-COF with a width diameter of about 15 nm, as shown in TEM images in Figure 3d–f.

**Figure 4:**
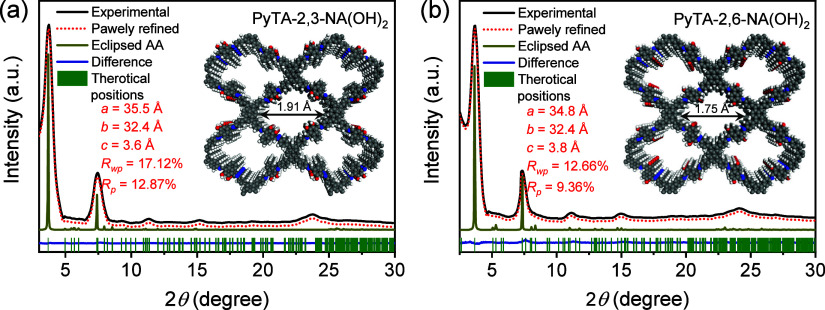
(a, b) Powder XRD (PXRD) patterns of PyTA-2,3-NA(OH)_2_ and PyTA-2,6-NA(OH)_2_ HO-COFs (black). Pawley refinement (red) was compared with the simulated PXRD pattern of eclipsed A–A stacking modeling (olive yellow).

The lattice structure of the HO-COFs was determined by powder X-ray diffraction (PXRD) (Figure 4). The PXRD patterns of PyTA-2,3-NA(OH)_2_ and PyTA-2,6-NA(OH)_2_ HO-COFs reveal strong diffraction peaks at 2θ = 3.74 and 3.67°, respectively, which are assignable to the reflection from the (110) plane (Figure 4a,b). In addition, the diffraction peaks at 2θ = 5.51, 7.64, and 23.85° for PyTA-2,3-NA(OH)_2_ and 5.50, 7.63, and 24.30° for the PyTA-2,6-NA(OH)_2_ HO-COF are characteristic of the (200), (210), and (001) reflection planes, respectively. Fractional atomic coordinates for the unit cell of PyTA-2,3-NA(OH)_2_ and PyTA-2,6-NA(OH)_2_ HO-COFs with A–A stacking are tabulated in Tables S1 and S2. The experimental PXRD pattern fits closely with the simulated pattern of A–A stacking rather than AB stacking in terms of the positions and intensities of the peaks. The eclipsed A–A stacking is used for the Pawley refinement, resulting in PXRD patterns that are very close to the experimental data. The unit cell parameters are in the ranges of *a* = 35.5 Å, *b* = 32.4 Å, and *c* = 3.6 Å for PyTA-2,3-NA(OH)_2_ and *a* = 34.8 Å, *b* = 32.4 Å, and *c* = 3.8 Å for PyTA-2,6-NA(OH)_2_ (α = β = γ = 90° for both HO-COFs), which are very close to the predictions, with good agreement factors, achieving weight-profile *R*-factor *R*_wp_ = 17.12% and weight-profile *R*-factor *R*_p_ = 12.87% and *R*_wp_ = 12.66% and *R*_p_ = 9.36% for PyTA-2,3-NA(OH)_2_ and PyTA-2,6-NA(OH)_2_, respectively. The interlayer π–π stacking distance between the COF layers was calculated and found to be 3.73 and 3.66 Å, from the *d* spacing of the (001) plane (Tables S1–S3).

**Figure 5:**
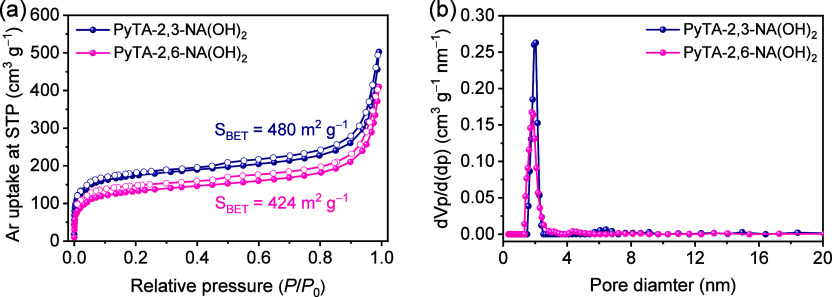
(a, b) Ar adsorption–desorption isotherms and pore size distributions of the pyrene-based HO-COFs.

The decomposition temperatures of 10 wt % (*T*_d10%_) for PyTA-2,3-NA(OH)_2_ and PyTA-2,6-NA(OH)_2_ HO-COFs are 446 and 476 °C, respectively (Figure S9). The carbonized residue of the COFs is above 60% at 800 °C. The presence of pyrene units within the COFs dramatically improves their thermal stability due to their relatively high degree of π stacking.^14^ PyTA-2,3-NA(OH)_2_ and PyTA-2,6-NA(OH)_2_ HO-COFs exhibit higher char yields due to the covalent linkages and high cross-linking density of the networks (Figure S9 and Table S4). The porosities of the HO-COFs were assessed by using argon (Ar) adsorption–desorption isotherms at 87 K (Figure 5a). Fully reversible isotherms are observed, exhibiting a rapid Ar uptake at low relative pressures (*P*/*P*_0_ < 0.01). The isotherms of both HO-COF nanofibers exhibit a steep rise at very low relative pressure, indicating that the HO-COFs are substantially microporous materials. At higher *P*/*P*_0_, the adsorbed volume of Ar gas slightly increases, displaying a type I isotherm according to IUPAC classification, implying a high degree of microporosity.^55,57,58^ As seen from Figure 5a, the PyTA-2,3-NA(OH)_2_ HO-COF isotherm shows the highest apparent Brunauer–Emmett–Teller (BET) surface area (480 m^2^ g^–1^), which is higher than that obtained for the PyTA-2,6-NA(OH)_2_ HO-COF (424 m^2^ g^–1^) (Figure 5a). The obtained HO-COF has a relatively low BET surface area compared to previously reported studies,^59,60^ likely due to its condensed molecular structure for its naphthalene subunits and longer connecting structure.

The pore size distributions of HO-COFs were estimated by using the quenched solid density functional theory (QSDFT) from the adsorption branches of the Ar isotherms (Figure 5b). The QSDFT-estimated pore sizes distributions demonstrate that the obtained PyTA-2,3-NA(OH)_2_ and PyTA-2,6-NA(OH)_2_ HO-COFs are porous in nature with measured pore diameters of 2.05 and 1.81 nm, respectively (Figure 5b and Table S3), which are much consistent with the determined theoretical pore sizes for PyTA-2,3-NA(OH)_2_ (1.91 nm) and PyTA-2,6-NA(OH)_2_ (1.75 nm) HO-COFs (Figure 4).

### 3.2 QCM Sensing Performance toward EDA Vapors

Driven by the high, microporous nature and the directionality of the tailored hydroxyl groups, our tailor-made HO-COFs, as a novel class of porous materials, demonstrated distinguished sensing activity and selectivity for EDA gas. Their microporous nature is accessible to the targeted guest molecule. As a proof of concept, the 2,3-dihydroxynaphthalene (2,3-NA(OH)_2_) and 2,6-dihydroxynaphthalene (2,6-NA(OH)_2_) monomers were employed as sensing materials for the detection of EDA vapor by the QCM technique (Figure S10a–d). The fabricated 2,3-NA(OH)_2_ and 2,6-NA(OH)_2_-based QCM sensors were exposed to 100 ppm vaporized EDA and other vapor-hazardous deleterious substances in the air at ambient conditions. The time-dependent frequency shift (Δ*F*) of the QCM sensor was successfully recorded (Figure S10c). The fabricated 2,3-NA(OH)_2_- and 2,6-NA(OH)_2_-based QCM sensors demonstrate the highest sensing uptake of Δ*F* = 150.1 and 101.4 Hz, respectively, for an EDA vapor of 100 ppm. Further, the sensing uptake attained for organic and water vapors (all at 100 ppm) is significantly lower than that for vaporized EDA (Figure S10c). Remarkably, the fabricated 2,3-NA(OH)_2_-coated QCM sensor exhibits a high selectivity of approximately 67% toward EDA vapor, compared to the 2,6-NA(OH)_2_-modified sensor, which shows 55% selectivity under the same conditions (Figure S10d). Based on these results, it is concluded that the directionality of neighboring hydroxyl groups facing the same direction on the surface of 2,3-NA(OH)_2_ significantly improves EDA sensitivity and selectivity, compared to 2,6-NA(OH)_2_, which contains surface hydroxyl groups facing the opposite direction.

**Figure 6:**
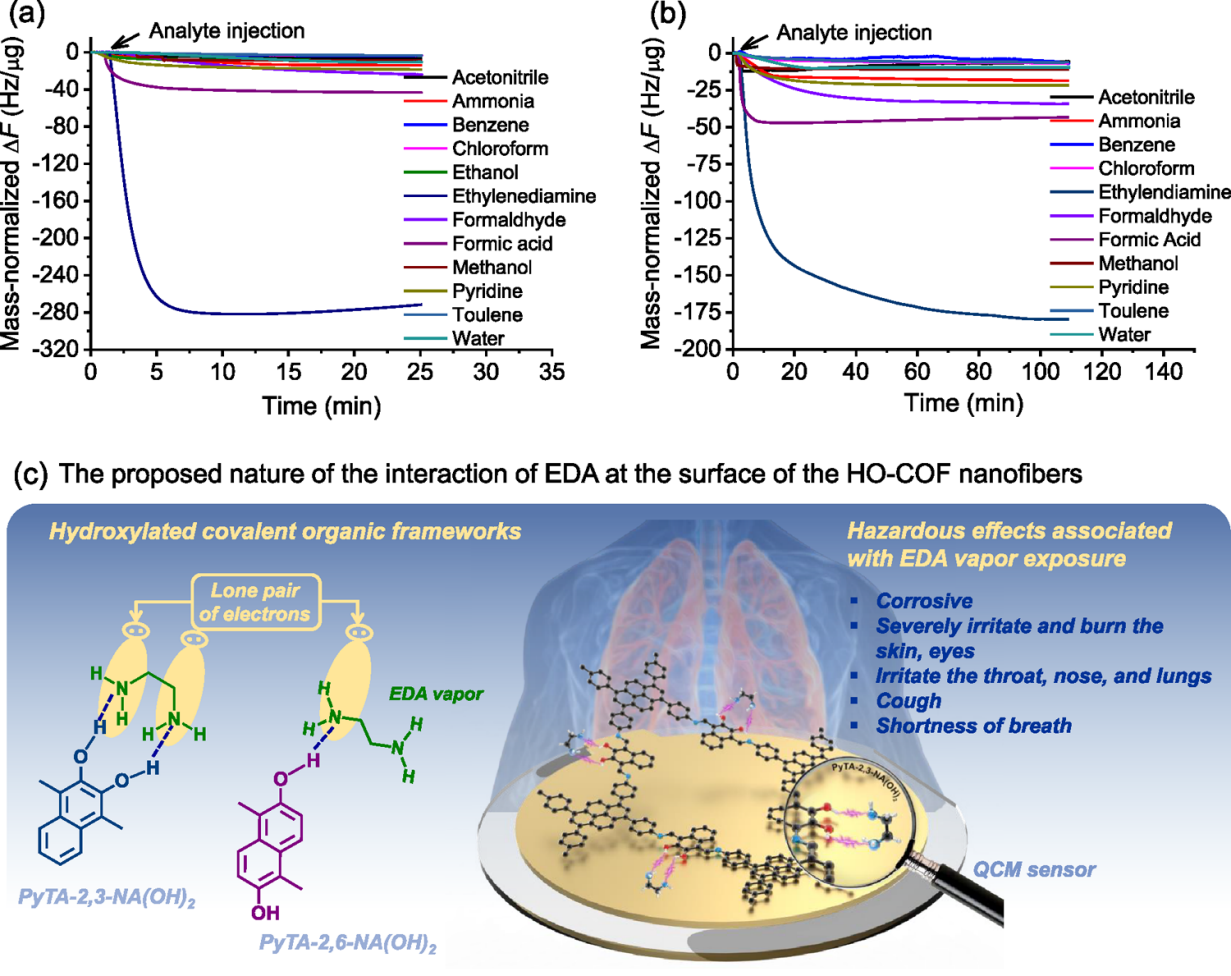
Mass-normalized time-dependent Δ*F*s of the QCM sensor upon exposure to a wide variety of VOCs using (a) PyTA-2,3-NA(OH)_2_ and (b) PyTA-2,6-NA(OH)_2_ HO-COFs at ambient temperature and pressure. The injected concentration of VOCs is 100 ppm. (c) Proposed adsorption mechanism for the interaction of EDA at the surface of HO-COF nanofibers.

To demonstrate the practical applicability of the synthesized HO-COFs, a QCM sensing system was fabricated using structure-induced selective PyTA-2,3-NA(OH)_2_ and PyTA-2,6-NA(OH)_2_ HO-COFs nanofibers as coating layers for the selective detection of EDA vapor (Figure S11). EDA is a corrosive chemical, and contact with it can burn, irritate, and cause severe skin and eye damage. Inhalation of EDA can irritate the throat, nose, and lungs, resulting in coughing or shortness of breath.^35^ Hence, owing to the detrimental effects of EDA on human health and the environment, this study presents an outstanding HO-COF structure-induced selective and stable QCM chemical sensor for the rapid and real-time monitoring of EDA vapors. This work establishes HO-COF-based QCM sensor discrimination of EDA in the vapor phase by PyTA-2,3-NA(OH)_2_ and PyTA-2,6-NA(OH)_2_ nanofibers using the QCM sensor technique (Figure 6). The relationship between Δ*F* (Hz) and the mass per unit area (Δ*m*, g cm^–2^) deposited on the Au electrode of the QCM sensor at a fundamental resonant frequency, *F*_0_, is described in eqs S1, S2 (see Supporting Information). The masses of the deposited PyTA-2,3-NA(OH)_2_ and PyTA-2,6-NA(OH)_2_ HO-COFs on the QCM electrodes were calculated to be 3.18 and 3.64 μg, respectively, based on eq S3. All the recorded frequencies were normalized by mass to determine sensitivity and selectivity.

Preliminary sensing tests with an uncoated Au electrode revealed that it had insignificant responses to EDA vapor. In this context, two hydroxyl-based COF nanofiber-modified QCM sensors of PyTA-2,3-NA(OH)_2_ and PyTA-2,6-NA(OH)_2_ nanofibers were fabricated and exposed to vaporized EDA. These fabricated QCM-based sensors are composed of porous HO-COFs having abundant hydrogen bonding sites with selectively induced adsorption properties toward EDA vapors due to the location of the hydroxyl groups on the surface of the COF. Experiments with QCM sensor were conducted to assess the gas sensing activity and selectivity of HO-COF nanofibers toward volatile EDA vapors. From Figure 6, the HO-COF-based fabricated QCM sensor was subjected to 100 ppm of the vaporized EDA. The time-dependent frequency shift (Δ*F*) of the QCM sensor was recorded, and the corresponding frequency dramatically deceased immediately after EDA injection, owing to the significant adsorption uptake of EDA molecules by PyTA-2,3-NA(OH)_2_ (Δ*F* = 281.2 Hz) and PyTA-2,6-NA(OH)_2_ (Δ*F* = 179.6 Hz) HO-COFs. After that, the frequency of the EDA-adsorbed HO-COF-based QCM sensor gradually returned to its initial values upon desorption of the volatile EDA vapor by using high-purity N_2_ gas (Figure S12). The PyTA-2,3-NA(OH)_2_ HO-COF (Δ*F* = 281.2 Hz) sensitivity for volatile EDA is outstanding, indicating its potential as a selective EDA sensor. Its response is 1.6 times higher than that of the PyTA-2,6-NA(OH)_2_ HO-COF (Δ*F* = 179.6 Hz) (Figure 6a,b), although their morphologies, BET surface areas and pore size distributions barely differ. Thus far, sufficiently exposed neighboring hydroxyl groups facing the same directions on the surface of the PyTA-2,6-NA(OH)_2_ HO-COF play a key role in interactions with EDA, leading to stronger hydrogen-bonding interactions (Figure 6c).

The presence of surface-abundant neighboring hydroxyl groups facing the same direction on the PyTA-2,3-NA(OH)_2_ HO-COF facilitates strong hydrogen bonding interactions with both (−NH_2_) groups of EDA, whereas PyTA-2,6-NA(OH)_2_ binds with EDA through only one OH group (DFT study about the adsorption mechanism is available in Section 3.4), as shown in Figure 6c. This demonstrates unequivocally how exposed hydrogen bonding sites arranged in the same direction can improve the sensing activity by detecting and quantifying a particular guest molecule. This arrangement reflects a higher base polarity, which leads to stronger polar–polar and hydrogen bonding interactions with the hydroxyl sites of the PyTA-2,3-NA(OH)_2_ and PyTA-2,6-NA(OH)_2_ HO-COFs.

**Figure 7:**
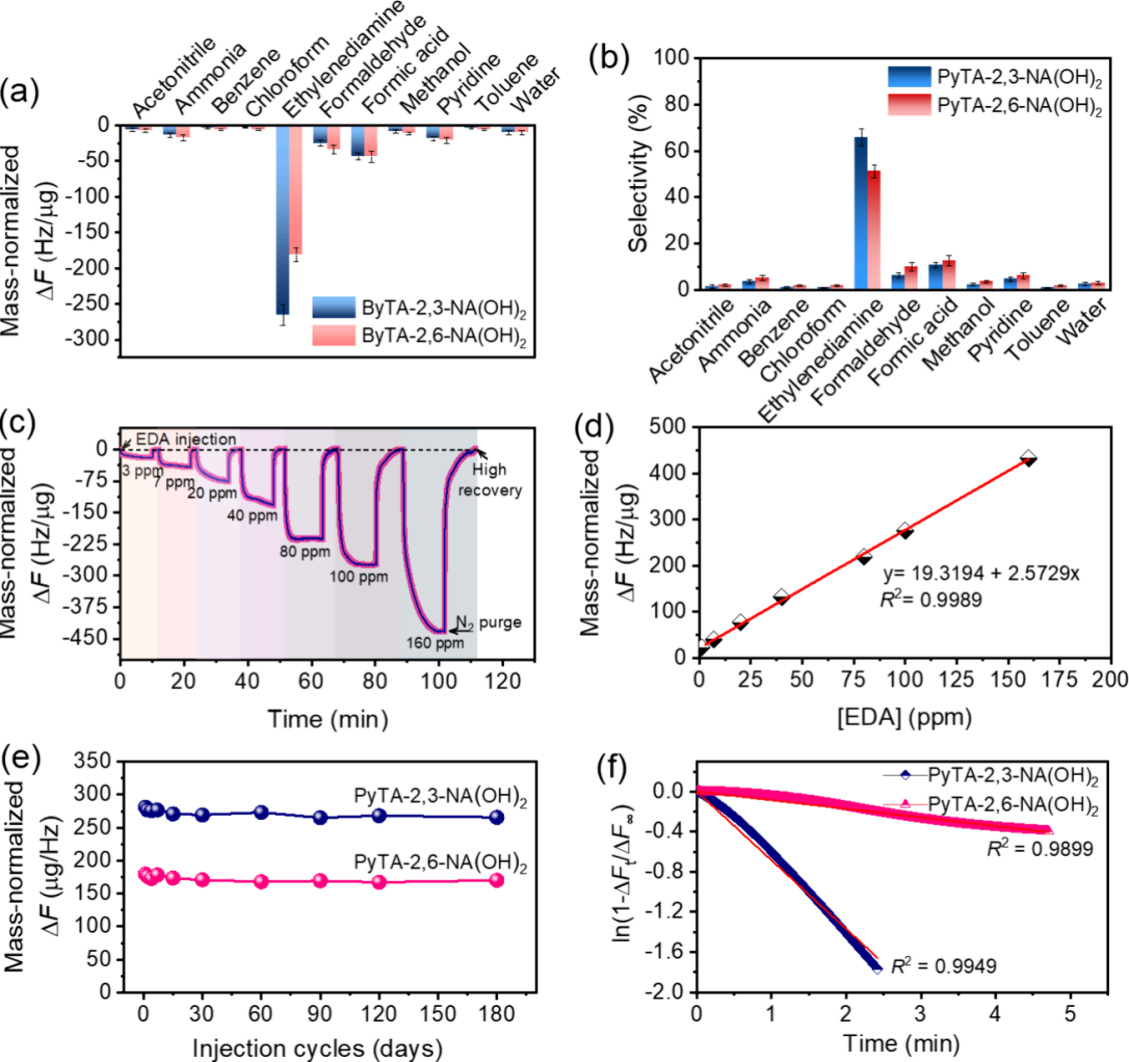
(a) Mass-normalized Δ*F*s and (b) selectivity test of PyTA-2,3-NA(OH)_2_ and PyTA-2,6-NA(OH)_2_-based QCM sensors toward a chemical-vapor analyte at room temperature. (c) Real-time response–recovery curves of the PyTA-2,3-NA(OH)_2_-modified QCM sensor at ambient temperature for various EDA vapor concentrations and (d) corresponding calibration curve. (e) Mass-normalized Δ*F* for the long-term stability test of HO-COF-based QCM sensor obtained by injection of EDA over a 6-month period. (f) Plot of ln(1 – Δ*F*_*t*_/Δ*F*_∞_) versus time, *t*, for the uptake of EDA vapor by the HO-COFs. Frequencies were recorded after injection of the chemical-vapor analyte at 100 ppm.

The exceptional ability of PyTA-2,3-NA(OH)_2_- and PyTA-2,6-NA(OH)_2_-modified QCM sensor to selectively discriminate EDA vapor from other VOCs and water vapor (moisture) is further demonstrated by selectivity studies (Figure 7). PyTA-2,3-NA(OH)_2_ and PyTA-2,6-NA(OH)_2_ HO-COFs were subsequently employed as selective active materials for EDA sensing in the presence of a variety of different chemical-vapor analytes, employing the QCM technique (Figure 7a). In particular, the PyTA-2,3-NA(OH)_2_-coated QCM sensor exhibits the most significant adsorption uptake of Δ*F* = 281.2 Hz when exposed to 100 ppm EDA vapor. This is significantly higher than the adsorption uptake obtained for water vapor (Δ*F* = 10.2 Hz) and organic vapors (all at 100 ppm), including ammonia (Δ*F* = 14.1 Hz), pyridine (Δ*F* = 18.6 Hz), formaldehyde (Δ*F* = 25.4 Hz), formic acid (Δ*F* = 43.1 Hz), methanol (Δ*F* = 9.0 Hz), acetonitrile (Δ*F* = 6.0 Hz), trichloromethane (Δ*F* = 4.0 Hz), benzene (Δ*F* = 4.2 Hz), and toluene (Δ*F* = 3.7 Hz) (Figure 6a). The PyTA-2,6-NA(OH)_2_-coated QCM sensor exhibits the highest adsorption uptake of Δ*F* = 179.6 Hz when exposed to 100 ppm vaporized EDA. This is significantly higher than the sensing uptake of water vapor (Δ*F* = 10.0 Hz) and organic vapors (all at 100 ppm), including ammonia (Δ*F* = 17.7 Hz), pyridine (Δ*F* = 21.1 Hz), formaldehyde (Δ*F* = 34.2 Hz), formic acid (Δ*F* = 43.8 Hz), methanol (Δ*F* = 11.8 Hz), acetonitrile (Δ*F* = 7.4 Hz), trichloromethane (Δ*F* = 5.9 Hz), and benzene (Δ*F* = 6.1 Hz) (Figure 6a). The fabricated HO-COF-based QCM sensor was exposed to EDA vapor and other organic vapors (all injected analytes at 100 ppm), as shown in Figure 7b, to assess their selectivity. It has been noted that a variety of tri- and tetra-amino-functionalized monomers, including 1,3,5-tris(4-aminophenyl)triazine and *N*,*N*,*N*′,*N*′-tetrakis(4-aminophenyl)-1,4-phenylenediamine can be used to synthesize COFs by interacting with 2,3-NADC and 2,6-NADC.^46,61^ Nevertheless, the framework structure of these monomers includes nitrogen heteroatoms, which may reduce their selectivity for EDA vapor. For instance, the selectivity toward EDA is anticipated to be dramatically reduced if formic acid vapors have been present as an interfering gas or a chemical-vapor analyte, as formic acid can form a strong hydrogen bonding interaction with the *N*,*N*,*N*′,*N*′-tetrakis(4-aminophenyl)-1,4-phenylenediamine-based COF.^46^ We, therefore, set out to design a structure-induced enhanced selectivity to EDA vapors by changing the directionality of hydroxyl groups to improve the sensing affinity and selectivity to EDA vapor via a strong hydrogen bonding interaction between EDA and hydroxyl groups inside the COFs (Figure 6c). Thus, the pyrene unit was selected for our HO-COF-based selective sensor since it does not contain heteroatoms. The results confirm the distinguished selectivity of the PyTA-2,3-NA(OH)_2_ HO-COF-based sensor toward EDA vapor in air at ambient conditions, regardless of the presence of interfering chemical analytes, as a result of the electron-withdrawing nature of the neighboring hydroxyl group on the surface of the COF. From the data in Figure 7b, the selectivity values of the HO-COF-modified QCM sensor toward EDA vapor can be calculated using eq 1.
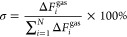
1where  represents the response of the QCM sensor for a particular vaporized analyte. As shown in Figure 7b, the PyTA-2,3-NA(OH)_2_ HO-COF-coated QCM sensor shows a higher selectivity of approximately 65% to EDA vapor compared to that of the PyTA-2,6-NA(OH)_2_ HO-COF, which exhibits 51% selectivity at ambient conditions. These findings imply that the presence of the neighboring hydroxyl groups facing the same direction on the surface of PyTA-2,3-NA(OH)_2_ is a key factor in enhancing its sensing sensitivity and selectivity, compared to the PyTA-2,6-NA(OH)_2_ HO-COF, which has the surface hydroxyl groups facing opposite directions.

It has been noted that detecting a particular chemical-vapor analyte in a humidified atmosphere may result in a drift in the selectivity of the operating sensor. Therefore, the effect of humidity (water moisture) on the sensing response toward EDA vapor should be considered if the sensor is utilized in a workplace environment. The magnitude of the gas sensor response increases with the injection concentration of EDA. However, when pure water was used as the vapor source, the QCM sensor response was minimal (Figures 6 and 7). Figure 7 displays the sensor responses of EDA vapor at the same concentration of 100 ppm compared to water vapor. The PyTA-2,3-NA(OH)_2_ HO-COF sensor selectively responds to EDA vapor (Δ*F* = 281.2 ± 14.7 Hz) approximately 28 times greater than it responds to water vapor (Δ*F* = 10.2 ± 2.5 Hz). In contrast, the response of the PyTA-2,6-NA(OH)_2_ HO-COF to EDA vapor (Δ*F* = 179.6 ± 9.8 Hz) is about 18 times higher than its response to water vapor (Δ*F* = 10.0 ± 2.9 Hz). This indicates that, although a high water content in the working vessel may cause a response, the interference response from water vapor is negligible compared to the gas response caused by EDA vapor. The HO-COF nanofiber-based gas sensor with abundant exposed active neighboring hydroxyl groups side-by-side (C–OH) allows for an improved sensor response to 100 ppm EDA vapors but not to water at the same concentration. Since PyTA-2,3-NA(OH)_2_ nanofiber has significant nanoporosity and a large surface area, it necessarily adsorbs small polar molecules, such as H_2_O. The sensing response of water is dramatically low, with a selectivity reaching 2.5%, due to weak physical adsorption.

This superior sensing performance for EDA over other VOCs is due to the strong hydrogen bonding interactions between the two close neighboring hydroxyl groups side-by-side of PyTA-2,3-NA(OH)_2_ with both (−NH_2_) of EDA (NH_2_(CH_2_)_2_NH_2_). The remarkable affinity of the PyTA-2,3-NA(OH)_2_ HO-COF toward EDA vapor is supported by the density functional theory (DFT) study to understand the adsorption mechanism (Section 3.4). This study suggests that the strong affinity of the hydroxylated COFs for basic EDA vapor is caused by the strong chemisorption of EDA molecules on the surface of PyTA-2,3-NA(OH)_2_ nanofibers through hydrogen bonding interactions of the two neighboring OH-functional groups having the same direction side-by-side with both (−NH_2_) of EDA. The EDA molecule has a strong Lewis basicity with a high electron cloud density, and its nitrogen atoms contain four lone pairs of electrons. Therefore, the hydroxyl groups of the HO-COF can act as the adsorption sites of EDA for chemisorption and promote charge transfer, resulting in the frequency change and, thus, the gas sensing response in the HO-COF. Furthermore, the high nanoporosity of the as-prepared HO-COF resulted in a high BET surface area and exposure of two abundant neighboring hydroxyl groups in the same direction as the target chemical-vapor analyte. These unique features contribute to the superior gas sensing capability of the as-prepared HO-COF nanofibers for EDA. The benzene rings and electron-withdrawing groups in the HO-COF structure significantly impact its gas sensing properties. The conjugation effect is present in the HO-COF framework nanofiber due to the conjugated system between the benzene rings and hydroxyl groups. The interaction between the electronic orbits of each atom suggests that the π-electrons around the atom are delocalized and freely migrate across atoms, leading to an equalized electron cloud density throughout the conjugated system. The oxygen atom of the hydroxyl group in the connecting anthracene units of HO-COF nanofibers has a higher electronegativity than the hydrogen atom, demonstrating the induction effect of electron withdrawal. As a result, oxygen connected to the benzene ring is partially negatively charged, and hydrogen is partially positively charged. The benzene rings that have been introduced exhibit both the electron-donating conjugation effect and the electron-absorption induction effect. The electron cloud distribution of the conjugated system is more uniform, resulting in a decrease in the negative charge of the oxygen atom connected to the benzene ring due to the electron-withdrawing nature of benzene, a shorter bond length between the oxygen atom and the benzene ring, and an increase in the positive charge of the hydrogen atom. The electron affinity and Lewis acidity of the hydroxyl group become much stronger, implying a significant increase in the ability to interact with amine groups of the EDA molecule and charge transfer for HO-COF nanofibers. It is evident from the gas sensing performance that PyTA-2,3-NA(OH)_2_ nanofibers exhibit a greater gas sensing response toward EDA, mainly as a result of the interaction between both amino groups of EDA molecules with the abundant two adjacent hydroxyl groups side-by-side that have the same directionality on the surface of PyTA-2,3-NA(OH)_2_ nanofibers. The gas sensing performance of PyTA-2,3-NA(OH)_2_ nanofibers toward EDA can be enhanced by lowering the density of the electron cloud on the hydroxyl group, increasing its Lewis acidity, all of which contribute to maintaining the stability of the molecular structure of COFs. Although pyridine, NH_3_, and EDA are basic deleterious chemical compounds, a more significant Δ*F* is observed for EDA compared to pyridine and NH_3_ (Figure 7a). EDA is more basic than pyridine and NH_3_, which can result in greater hydrogen bonding interactions with the neighboring hydroxyl groups on the surface of PyTA-2,3-NA(OH)_2_. Furthermore, the PyTA-2,3-NA(OH)_2_-modified QCM sensor shows a reduced sensitivity to vaporized chlorinated and aromatic hydrocarbons, such as trichloromethane, benzene, and toluene, with the lowest Δ*F*s and only weak responses at 4.0, 4.2, and 3.7 Hz. These findings suggest that these VOCs favor physisorption over chemisorption.

The dynamic sensing behavior of EDA vapors during sequential injection into the glass vessel at different concentrations of 3–160 ppm was studied using the PyTA-2,3-NA(OH)_2_-coated QCM sensor (Figure 7c). The adsorption uptake of EDA by the PyTA-2,3-NA(OH)_2_ HO-COF was demonstrated by a drastic decrease in frequency, signifying the uptake of vapors (Δ*F* = 19.8 ± 3.2 Hz) when the injected concentration reached 3 ppm into the working cell. With the increase in the EDA vapor concentration, the sensing uptake of EDA vapors is enhanced remarkably, recording a Δ*F* of 433.6 ± 17.2 Hz at 160 ppm. The sensing response of the PyTA-2,3-NA(OH)_2_ HO-COF increases linearly with the injection of EDA, as shown by the calibration graph of Δ*F*s against the EDA concentration (Figure 7d). Based on the calibration graph, the fabricated sensor exhibits a high average detection sensitivity, which is the slope of the calibration graph of 2.57 Hz ppm^–1^ toward EDA vapors with an acceptable precision of 2.49% as a relative standard deviation with the PyTA-2,3-NA(OH)_2_ HO-COF. The distinguished sensitivity of the fabricated sensor enables the use of the PyTA-2,3-NA(OH)_2_ HO-COF to detect vaporized EDA with a low limit of detection (LoD) of 2.9 ppm at ambient temperature and pressure. The LoD was determined using the calibration curve (*y* = 19.3194 + 2.5729*x*; *R*^2^ = 0.9989) based on the data presented in Figure 7d and calculated according to eq 2.
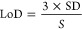
2where SD and *S* describe the standard deviation and slope of the calibration curve of the sensor response, respectively. This sensor can detect EDA vapor at concentrations as low as 2.9 ppm, which is significantly lower than the Occupational Safety and Health Administration’s (OSHA) human olfactory threshold limit in the workplace of 10.0 ppm for an 8 h work shift, which is the level at which airborne EDA irritates the eyes, nose, and throat of the most sensitive individuals.

**Figure 8:**
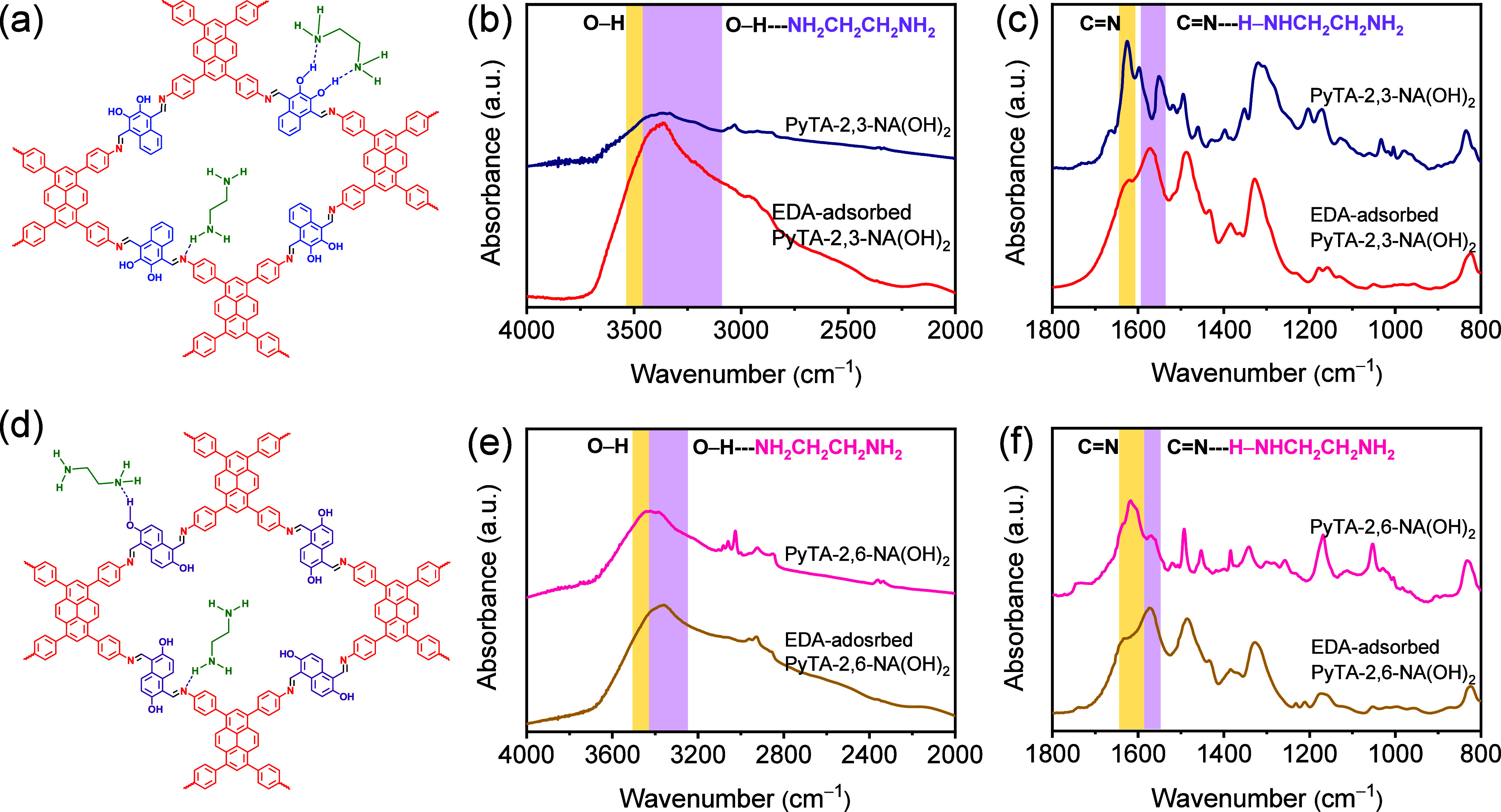
(a, d) Representation of hydrogen bonding interactions between PyTA-2,3-NA(OH)_2_ and PyTA-2,6-NA(OH)_2_ HO-COFs and EDA molecules. FTIR spectra of (b, e) the EDA-adsorbed PyTA-2,3-NA(OH)_2_ HO-COF and (c, f) the EDA-adsorbed PyTA-2,6-NA(OH)_2_ HO-COF measured at room temperature.

Although attention has been paid to developing EDA vapor detection methodologies based on optical spectroscopies, conductometry, gas chromatography–mass spectrometry, high-performance liquid chromatography based on previously reported materials, including various thiol-substituted compounds,^38^ MoO_3_ nanoribbons modified with rGO nanosheets,^40^ porphyrin complexes,^62,63^ and Y-type zeolite-based terbium-acetylacetonate complexes,^64^ our fabricated QCM sensor using PyTA-2,3-NA(OH)_2_ provides a cost-effective, reliable, rapid, and highly sensitive method for detecting the small mass change at the surface of the electrode in the nanogram range at ambient temperature and pressure. In addition, this HO-COF-based sensor can be adapted for the on-site application of EDA sensing with distinguished selectivity by carefully controlling the directionality of hydroxyl groups and the long-term stability, which meets the requirements in real sensing applications. The sensor response of PyTA-2,3-NA(OH)_2_ is either much higher or relatively lower compared to porphyrin complexes and their composites (Table S5). Although some reports have achieved a somewhat lower LoD, their selectivities were not as good as our representative PyTA-2,3-NA(OH)_2_-modified QCM sensor in this study.^62,63^ Importantly, a suitable balance between the surface area, porosity, and the tailored design of the active function group should be taken into account to get high sensing activity and selectivity for the chemical-vapor analyte. The surface area of PyTA-2,3-NA(OH)_2_ is significantly higher than that of previously reported materials utilized for EDA sensing. Therefore, the overall response is remarkable due to the synergetic cooperation between the directionality of tailored hydroxyl groups, high specific surface area (480 m^2^ g^–1^), and the microporous nature of the HO-COF, which facilitate the fast diffusion uptake of EDA vapor. As seen from Table S5, other reported materials cannot simultaneously possess many textural advantages. Practically, enhancing one functionality negatively impacts other properties, eventually reducing the sensing performance.

Furthermore, the reproducibility of the sensor is an essential concern for assessing its efficacy. The reproducibility of the PyTA-2,3-NA(OH)_2_-modified QCM sensor was evaluated in terms of the base frequency after achieving complete adsorption of EDA molecules. As demonstrated in Figure 7c, the PyTA-2,3-NA(OH)_2_ nanofiber-based QCM sensor recovered up to 99.1% of its initial frequency rapidly in 2–3 min after being purged with high-purity N_2_ gas that passed through the working vessel, and the chemisorbed EDA molecules were rapidly desorbed. The results of cycling tests clearly show the distinguished reproducibility of the HO-COF-based QCM sensor for EDA vapor, demonstrating no deviation from the baseline (Figure 7c). Even at high injection concentrations, PyTA-2,3-NA(OH)_2_ nanofibers show distinguished reproducibility during alternate exposure and removal of EDA with an approximately 0.9% decrease in Δ*F* and no observable deterioration. After demonstrating the high sensing activity and selectivity of HO-COF-modified QCM sensors toward EDA (100 ppm), their long-term stability over 6 months was evaluated, which is an essential concern for chemical gas sensors and electronic nose devices. The sensing responses of the COF-based sensors toward EDA (100 pm) were tested for 6 months and are depicted in Figure 7e, demonstrating their good long-term stability. The data show that the Δ*F* of PyTA-2,3-NA(OH)_2_ or PyTA-2,6-NA(OH)_2_ HO-COF-based sensors decreases by about 6.4 and 6.8%, respectively, under continuous injection of EDA, suggesting their excellent cycling performance and good long-term stability.

Real-time monitoring of Δ*F*s of QCM sensor coated either with the PyTA-2,3-NA(OH)_2_ or PyTA-2,6-NA(OH)_2_ HO-COF was used to investigate the adsorption kinetics of EDA, which contributes to understanding the improved sensing performance of the HO-COF. The experimental real-time responses (Δ*F*s) for EDA adsorption onto COF-coated QCM sensor can be fitted to the pseudo-first-order kinetic model (eqs S4–S6 in the Supporting Information), and the results are shown in Figure 7f. QCM sensing of EDA by PyTA-2,3-NA(OH)_2_ and PyTA-2,6-NA(OH)_2_ COFs follows a pseudo-first-order kinetic model (Figure 7f). Based on the plots of ln(1 – Δ*F*_*t*_/Δ*F*_∞_) against time, *t*, the pseudo-first-order kinetic rate constants (*k*_1_s) obtained from the linear regression of the slopes for PyTA-2,3-NA(OH)_2_ and PyTA-2,6-NA(OH)_2_ HO-COFs are 68.51 × 10^–2^ ± 0.00129 and 8.59 × 10^–2^ ± 0.00016 min^–1^, respectively (Figure 7f). Consistent with the order of sensitivity, the adsorption of EDA by PyTA-2,3-NA(OH)_2_ proceeds at an approximate 8.0 times higher uptake rate than that by the PyTA-2,6-NA(OH)_2_ COF, probably due to the relatively higher surface area and the direction of the abundant hydroxyl active groups of the former, which can let EDA molecules diffuse more easily inside the cavities and interact with the active metal sites through strong acid–base interactions. To assess the chemical stability of the PyTA-2,3-NA(OH)_2_ and PyTA-2,6-NA(OH)_2_ HO-COF-based sensors in alkaline environments, HO-COFs (60 mg) were immersed in aqueous solutions of KOH (1 M) and EDA (160 mg L^–1^) for 24 h, followed by filtration. Subsequently, the HO-COFs were characterized by using PXRD and FTIR (Figure S13). Clearly, the PXRD and FTIR measurements of KOH- and EDA-immersed PyTA-2,3-NA(OH)_2_ and PyTA-2,6-NA(OH)_2_ HO-COFs show no alterations in peak intensities or positions after immersing in KOH and EDA aqueous solutions (Figure S13), implying that HO-COF-based sensors demonstrate high chemical stability in alkaline media, making them highly durable for alternate chemical-vapor adsorption–desorption measurements.

### 3.3 Hydrogen Bonding Study of EDA at the Surface of HO-COF Nanofibers

The hydrogen bonding between HO-COF nanofibers and EDA was investigated by the UV–vis diffuse reflectance spectrum (UV–vis DRS), FTIR, PXRD, TGA, and BET measurements, as well as visual calorimetric assessment. The FTIR disks fabricated from PyTA-2,3-NA(OH)_2_ and PyTA-2,6-NA(OH)_2_ HO-COFs were subjected to EDA vapor for 1 min at ambient temperature, after which the spectra were recorded for the obtained EDA-adsorbed PyTA-2,3-NA(OH)_2_ and EDA-adsorbed PyTA-2,6-NA(OH)_2_ (Figure 8a–f and Figure S14). Figure 8b,c illustrates the room-temperature FTIR analyses of the EDA-adsorbed PyTA-2,3-NA(OH)_2_ within the ranges of 4000–2000 cm^–1^ (O–H stretching) and 1800–800 cm^–1^ (C=N stretching). In contrast, Figure 8e,f depicts room-temperature FTIR analyses of the EDA-adsorbed PyTA-2,6-NA(OH)_2_ in the same ranges. A prominent band at 3440 cm^–1^ in the FTIR spectrum of pure PyTA-2,3-NA(OH)_2_ is attributed to the free O–H groups. In contrast, the FTIR spectrum of the EDA-adsorbed PyTA-2,3-NA(OH)_2_ reveals a new characteristic band at 3363 cm^–1^, which is ascribed to the strong hydrogen bonding interactions between the O–H groups in PyTA-2,3-NA(OH)_2_ and the amine (−NH_2_) groups of EDA (Figure 8a,b). Furthermore, a band observed at 1625 cm^–1^ is characteristic of the unbonded C=N groups, which shifted to 1572 cm^–1^ upon exposure to EDA vapor, indicating the formation of hydrogen bonding interactions between the imine (C=N) groups in PyTA-2,3-NA(OH)_2_ and the amine (−NH_2_) groups of EDA (Figure 8a–c). A similar phenomenon was observed for a phenylenediamine-based covalent organic framework (TPDA-TPB COF) when exposed to formic acid gas, forming hydrogen bonding interactions with the outermost layer of the TPDA-TPB COF.^46^

The FTIR spectrum of PyTA-2,6-NA(OH)_2_ reveals a characteristic band at 3447 cm^–1^ assignable to the free O–H groups, whereas EDA-adsorbed PyTA-2,6-NA(OH)_2_ shows a new band at 3360 cm^–1^ corresponding to the hydrogen bonding interactions between the hydroxyl (O–H) groups in PyTA-2,6-NA(OH)_2_ and the amine (−NH_2_) groups of EDA (Figure 8d,e). Another band at 1626 cm^–1^ in the PyTA-2,6-NA(OH)_2_ spectrum can be assigned to free C=N groups. This band is blueshifted to 1571 cm^–1^ after exposure to EDA, suggesting strong hydrogen bonding interactions between the imine (C=N) groups and the amine (−NH_2_) groups of EDA (Figure 8d–f).

To further confirm the hydrogen bonding interactions between the HO-COFs and EDA, 70 mg of PyTA-2,3-NA(OH)_2_ and PyTA-2,6-NA(OH)_2_ HO-COFs were exposed to EDA vapor for 1 min at ambient temperature. Subsequently, UV–vis DRS, PXRD, TGA, and BET were recorded for the resulting EDA-adsorbed PyTA-2,3-NA(OH)_2_ and EDA-adsorbed PyTA-2,6-NA(OH)_2_ HO-COFs. Visual colorimetric assessment and naked-eye detection reveal that the dry PyTA-2,3-NA(OH)_2_ HO-COF color changed from reddish brown to black when exposed to EDA vapor. In contrast, a color change from orange to red was observed for the dry PyTA-2,6-NA(OH)_2_ HO-COF, demonstrating the high adsorption affinity of the HO-COFs toward EDA (Figure S15a,b). The absorption thresholds of the dry PyTA-2,3-NA(OH)_2_ and PyTA-2,6-NA(OH)_2_ HO-COFs were measured at 538 and 499 nm, respectively, as shown in Figure S15c,d. After exposure to EDA vapor, the absorption onsets are redshifted to longer wavelengths of 587 and 529 nm for EDA-adsorbed PyTA-2,3-NA(OH)_2_ and EDA-adsorbed PyTA-2,6-NA(OH)_2_, respectively. The hydrogen bond interactions that stabilize the excited state between the HO-COFs and EDA are responsible for the observed redshift.^65^ Also, the as-synthesized PyTA-2,3-NA(OH)_2_ and PyTA-2,6-NA(OH)_2_ HO-COFs exhibit band gaps of 1.99 and 2.19 eV, respectively (Figure S16a,b). These values dropped to 1.78 and 2.08 eV after exposure to the EDA vapor. As can be seen, exposure of HO-COFs to EDA vapor does not alter their crystallinity, as demonstrated by the fact that the diffraction patterns for each HO-COF remain unchanged regarding the consistent number, shape, and location (Figure S17). Ar adsorption–desorption isotherms at 87 K were also used for the porosity assessment of the HO-COF-based sensors after exposure to EDA vapor (Figure S18a). The EDA-adsorbed PyTA-2,3-NA(OH)_2_ exhibits a BET surface area of 334 m^2^ g^–1^, which is higher than that of EDA-adsorbed PyTA-2,6-NA(OH)_2_ (306 m^2^ g^–1^). The relative decrease in Ar uptake and surface areas of the EDA-adsorbed HO-COFs can be ascribed to pore blockage by EDA vapors, indicating the hydrogen bond interactions between the HO-COFs and EDA.^66^ From TGA measurements, the EDA-adsorbed PyTA-2,3-NA(OH)_2_ and EDA-adsorbed PyTA-2,6-NA(OH)_2_ exhibit weight losses of 13.3 and 11.2%, respectively, within the temperature range of 77–130 °C, which can be attributed to the detachment of the bonded EDA molecules (Figure S18b).^67^ Furthermore, the hydrophilicity of the HO-COFs was evaluated by measuring the water contact angle. The measured contact angles on the surface of the PyTA-2,3-NA(OH)_2_ and PyTA-2,6-NA(OH)_2_ HO-COFs were 75.1 and 86.9°, respectively, suggesting that the PyTA-2,3-NA(OH)_2_ HO-COF is more hydrophilic, thus demonstrating an enhanced sensitivity toward harmful EDA (Figure S19).

### 3.4 DFT Calculations of the Stability of EDA at the Surface of HO-COF Nanofibers

**Figure 9:**
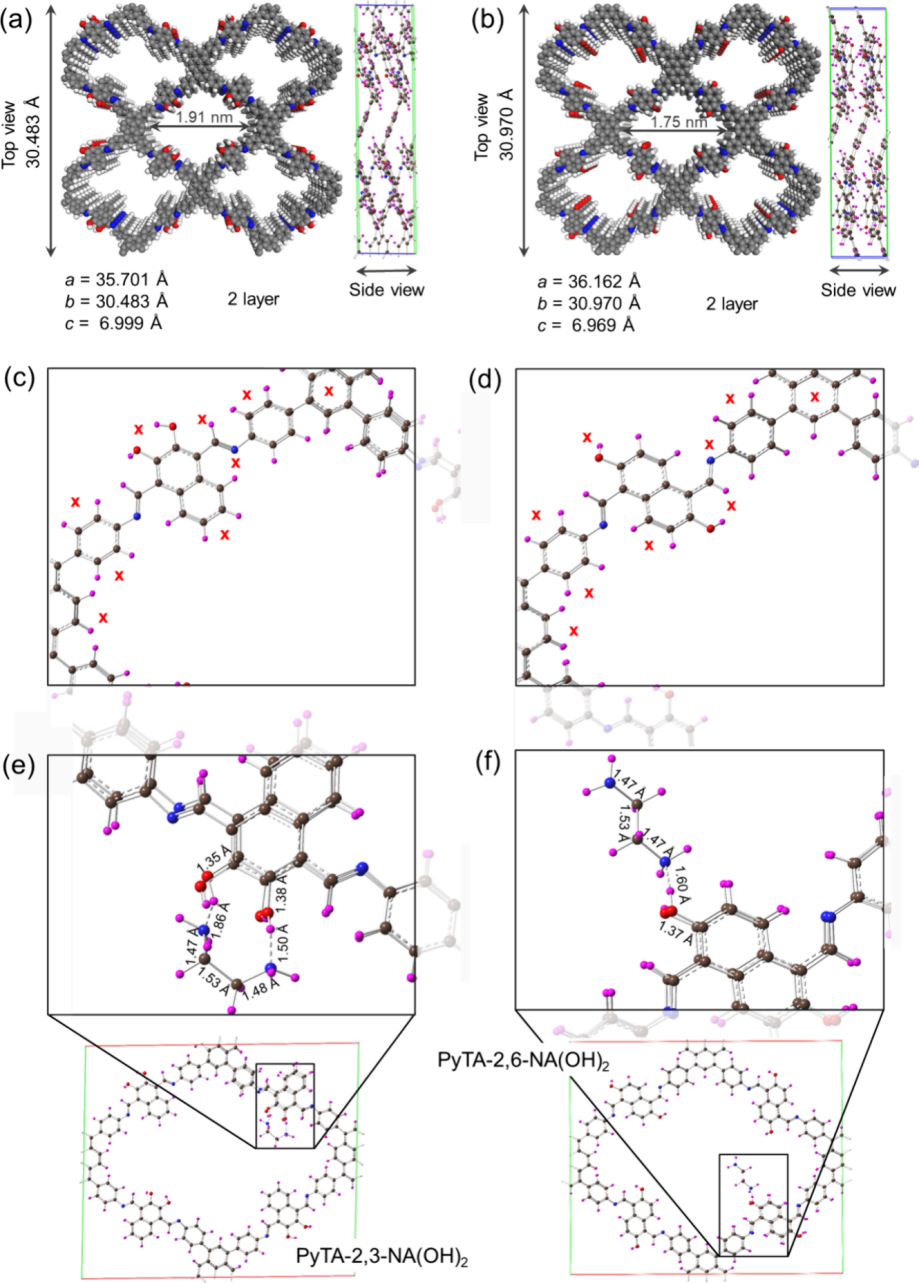
(a, b) Top and side views of the optimized structure, (c, d) selected probed adsorption sites of EDA, and (e, f) determined EDA adsorption sites on PyTA-2,3-NA(OH)_2_ and PyTA-2,6-NA(OH)_2_ HO-COFs. Dimensions are given in nanometers, and bond lengths are given in Ångstroms (Å).

Computational analysis was utilized to develop a model for calculating energy and understanding the nature of the interaction of EDA at the surface of the HO-COF nanofibers. The proposed model was built based on chemical composition data supplied by several characterization techniques. The most stable possible adsorption energy of 11 chemical analytes into the HO-COFs, PyTA-2,3-NA(OH)_2_ and PyTA-2,6-NA(OH)_2_ structures was calculated by DFT calculations using the Vienna ab initio simulation package (VASP).^68^ The structural optimization of the COF bulk unit cell of PyTA-2,3-NA(OH)_2_ and PyTA-2,6-NA(OH)_2_, which has a two-layered crystal structure, was performed with the Brillion Zone sampled using a 1 × 1 × 3 Monkhorst–Pack *k*-point grid. The predicted lattice parameters (*a*, *b*, and *c*) of PyTA-2,3-NA(OH)_2_ are *a* = 35.701 Å, *b* = 30.482 Å, and *c* = 6.999 Å, and of PyTA-2,6-NA(OH)_2_ are *a* = 36.162 Å, *b* = 30.970 Å, and *c* = 6.969 Å. The distance between the two layers in the unit cell model of both PyTA-2,3-NA(OH)_2_ and PyTA-2,6-NA(OH)_2_ is ∼3.5 Å, and their optimized structures are given in Figure 9a,b. It shows the top and side views of optimized molecular units of both HO-COFs. Both models have two distinct hydroxyl adsorption sites that bind the organic molecules. The adsorption site of the OH-functional group in the anthracene unit on the surface of PyTA-2,6-NA(OH)_2_ is one, and there are two neighboring OH groups side-by-side on PyTA-2,3-NA(OH)_2_ HO-COF nanofibers. The molecular structures of HO-COFs fully reflect the nature of the COF material (i.e., crystallinity). Like the one presented here, molecular models are useful for predicting the binding properties of smaller molecules, such as EDA (NH_2_–(CH_2_)_2_–NH_2_) in COFs. Several adsorption sites of adsorbed molecules were probed to identify the strongest adsorption site. Figure 9c,d demonstrates manually the selected initial adsorption sites of PyTA-2,3-NA(OH)_2_ and PyTA-2,6-NA(OH)_2_ HO-COFs. We additionally included about 18 randomly selected adsorption sites (and molecular orientations) for each HO-COF. The determined adsorption energy strength was confirmed with the Gaussian smearing method of 0.05 eV width. Figure 9e,f displays the configuration with the highest adsorption binding energy, as determined by eq S7, and clearly shows the EDA adsorption site with the strongest binding affinity, which is also supported by the binding distances between EDA and HO-COFs. The binding distances between NH_2_(CH_2_)_2_NH_2_ and HO-COFs are about 1.86 and 1.5 Å for PyTA-2,3-NA(OH)_2_ that have two neighboring OH-functional groups side-by-side and 1.6 Å for PyTA-2,6-NA(OH)_2_ having one exposed abundant surface OH group (Figure 9e,f and Table S6).

The calculated adsorption energies of all the 11 chemical analytes that bind with one OH-functional group in PyTA-2,6-NA(OH)_2_ and two neighboring OH groups in PyTA-2,3-NA(OH)_2_ HO-COFs, and the most stable configurations are identified and displayed in Figure 9e,f and Figure S20. Among all of the chemical analytes, the average adsorption energies for both models are approximately 32.1 and 25.8 kcal mol^–1^ for *cis*-EDA binding on the surface of PyTA-2,3-NA(OH)_2_ and PyTA-2,6-NA(OH)_2_ nanofibers, respectively. They are stronger for PyTA-2,3-NA(OH)_2_ nanofiber, with a minimum adsorption strength of 8.7 kcal mol^–1^. PyTA-2,6-NA(OH)_2_ adsorption energies range from 5.7 to 23.4 kcal mol^–1^. In this configuration, both N lone pairs of the EDA are hydrogen bonded to two neighboring OH groups side-by-side in PyTA-2,3-NA(OH)_2_, resulting in a higher adsorption energy. In the case of PyTA-2,6-NA(OH)_2_, the lone pair of N atoms of EDA binds to only the H of one OH group. Other interfering chemical-vapor analyte configurations exhibit much lower energy (Figure S20). This could indicate a generally stronger interaction between NH_2_(CH_2_)_2_NH_2_ and two close neighboring OH groups in PyTA-2,3-NA(OH)_2_ when more than one EDA molecule is adsorbed, as other adsorption sites are occupied.

It has been reported that larger macrocycles can enhance their adsorption energy by several eVs (kcal mol^–1^) in physisorbed systems due to a compounding effect that increases with size.^68,69^ On the other hand, the adsorption energies of macrocycles on chemically reactive surfaces range from −3 eV (69.5 kcal mol^–1^) to −5 eV (115.8 kcal mol^–1^).^70^ As a result, various energetic and geometric parameters should be addressed when determining the binding properties. Nonetheless, the adsorption energy of NH_2_(CH_2_)_2_NH_2_ on HO-COF nanofibers is relatively large, irrespective of size. The substantial negative value of the binding energy suggests a strong and exothermic interaction between adsorbed units, implying that binding can be either strongly physisorbed or weakly chemisorbed.^42^ EDA binds to OH-functional groups in PyTA-2,3-NA(OH)_2_ or PyTA-2,6-NA(OH)_2_ at sites where several weak N–H–O bonds can form between the N atom in NH_2_(CH_2_)_2_NH_2_ and the OH in the COF. Notably, the binding at the pyrene-like units is not as strong as N–H–O bonds. Significantly, NH_2_(CH_2_)_2_NH_2_ can form two N–H–O bonds with PyTA-2,3-NA(OH)_2_, whereas it can form only one bond in PyTA-2,6-NA(OH)_2_. This reflects the somewhat stronger binding to PyTA-2,3-NA(OH)_2_ nanofibers having abundant two close neighboring active hydroxyl groups that are in the same directionality, which can form a considerable number of hydrogen bonding interactions with both amino groups of EDA molecules in the same direction, suggests a slightly more robust binding to PyTA-2,3-NA(OH)_2_. Further, the shorter binding lengths between EDA and the PyTA-2,3-NA(OH)_2_ HO-COF also play a role. On the other hand, PyTA-2,6-NA(OH)_2_ nanofibers have abundant active hydroxyl groups that exist in the opposite direction, contributing to only one O–H–N bond with EDA. These DFT calculations utilizing the structure-dependent van der Waals correction yielded the structure shown in Figure 9a,b, consistent with the QCM-based sensor results.

Additionally, the adsorption energies were determined for interactions of EDA with neighboring OH groups in the adjacent layers in PyTA-2,3-NA(OH)_2_ and PyTA-2,6-NA(OH)_2_ HO-COFs (Figure S21). Computational calculations reveal that the adsorption energies of the interaction of the *cis-*EDA configuration with neighboring OH groups in the same layer and PyTA-2,3-NA(OH)_2_ are 32.1 and 27.9 kcal mol^–1^, respectively. On the other hand, the adsorption energy of the interaction of *cis-*EDA configuration with neighboring OH groups in the adjacent layers of PyTA-2,6-NA(OH)_2_ is 25.8 kcal mol^–1^, which is higher than the *trans*-EDA configuration of 23.4 kcal mol^–1^. The average adsorption energies were 27.9 and 25.8 kcal mol^–1^ for *cis*-EDA bonded with the OH groups of the adjacent layers in PyTA-2,3-NA(OH)_2_ and PyTA-2,6-NA(OH)_2_ nanofibers, respectively. Of the results, in comparison to the PyTA-2,6-NA(OH)_2_ HO-COF, EDA exhibited a stronger interaction with the PyTA-2,3-NA(OH)_2_ HO-COF. Furthermore, the potential interaction between EDA and the imine group of the HO-COF was also evaluated. The EDA molecules bind to the imine N groups of PyTA-2,3-NA(OH)_2_ and PyTA-2,6-NA(OH)_2_ at specific locations where weak N–H–N bonds may form between the N atom of the HO-COF and H–N in EDA (NH_2_(CH_2_)_2_NH_2_) with adsorption energies of 12.6 and 11.1 kcal mol^–1^, respectively (Figure 8a,d and Figure S22). These findings suggest that the PyTA-2,3-NA(OH)_2_ HO-COF exhibits a higher affinity to bind with the EDA molecules compared to PyTA-2,6-NA(OH)_2_.

## 4 Conclusions

In conclusion, this work has demonstrated the structure-induced selectivity of hydroxylated PyTA-2,3-NA(OH)_2_ and PyTA-2,6-NA(OH)_2_-based HO-COF nanofibers for advanced sensing applications of deleterious vaporized substances. The relationship between the structure and chemical compositions of HO-COFs and their gas sensing properties was carefully investigated, indicating distinguished selectivity for vaporized diamines. We confirmed that the tailor-made PyTA-2,3-NA(OH)_2_ with two close neighboring OH– groups in the same direction significantly demonstrated a rapid sensing response and distinguished selectivity toward EDA vapors, arising from the strong hydrogen bonding interactions with the NH_2_ groups of EDA, as investigated by a wide variety of chemical analysis techniques and DFT calculations. Visual colorimetric assessment and naked-eye detection revealed that the color of the HO-COF dramatically changed after exposure to EDA vapor, demonstrating the high adsorption affinity of the HO-COFs toward EDA. The measured contact angles revealed that the PyTA-2,3-NA(OH)_2_ HO-COF exhibited more hydrophilicity than the PyTA-2,6-NA(OH)_2_ HO-COF, indicating its enhanced sensitivity toward harmful EDA. The fabricated PyTA-2,3-NA(OH)_2_ HO-COF-modified QCM sensor showed a high sensitivity of 2.57 Hz ppm^–1^ and a low LoD reaching 2.9 ppm, allowing for selective detection of harmful EDA vapors over other interfering VOCs. The PyTA-2,3-NA(OH)_2_ COF with abundant exposed neighboring OH-functional groups facing the same direction exhibited 1.6 times higher sensitivity toward EDA vapor than the PyTA-2,6-NA(OH)_2_ HO-COF with OH groups in opposite directions. The sensor demonstrated remarkable long-term stability and distinguished selectivity to EDA in the presence of a wide variety of interfering chemical-vapor analytes and water. A pseudo-first-order kinetic model describes the adsorption of EDA vapors on the PyTA-2,3-NA(OH)_2_ HO-COF nanofiber; the adsorption rate is 8.0 times higher than that of PyTA-2,6-NA(OH)_2_ HO-COF nanofibers. EDA detection selectivity was attributed to (i) the abundance of active close neighboring OH-functional groups facing the same direction on the surface of HO-COF nanofibers and (ii) the thin porous nanofibrous coating layers induced by the sieving effect that provided better accessibility and enhanced OH adsorption sites for EDA molecules. DFT calculations supported the interaction mechanism between EDA and HO-COF nanofibers and showed that chemical and strong hydrogen bonding interactions are involved. Based on theoretical calculations, EDA exhibited a stronger interaction with the PyTA-2,3-NA(OH)_2_ compared to the PyTA-2,6-NA(OH)_2_ nanofiber HO-COF. This tailor-made structure-induced selective HO-COF-based sensor provides a straightforward, low-cost method for improving sensor performance in high sensitivity and selectivity for vapor discrimination in smart electronic noses.

## Supporting Information Available

Additional experimental details on the synthesis and characterization of HO-COFs; fractional atomic coordinates data; QCM gas sensor setup; mass-normalized time-dependent frequency change data analysis; visual colorimetric assessment and spectroscopic measurements of HO-COFs before and after exposure to alkaline solutions and EDA vapor; computational calculations; summary of reported studies on EDA sensing (PDF)
