# Supplementary material for: Structure-Induced Selectivity of Hydroxylated Covalent Organic Framework Nanofibers for Advanced Sensing Applications: An Experimental and Density Functional Theory Study
Source: ACS Appl Mater Interfaces. 2025 Apr 26;17(18):27188–203. doi: 10.1021/acsami.5c03407 (PMC12067381; doi:10.1021/acsami.5c03407)
Supplement: Supplementary file 1 — am5c03407_si_001.pdf [file am5c03407_si_001.pdf]

Supporting Information

Structure-Induced Selectivity of Hydroxylated Covalent Organic Framework Nanofibers for Advanced Sensing Applications: An Experimental and Density Functional Theory Study

Nagy L. Torad,[a,b,c] Tzu-Ling Yang,[d] Moustafa A. Darwish,[e] Putikam Raghunath,[f] Ahsanulhaq Qurashi,[a,b] Lamiaa Reda Ahmed,[d,g] Ming-Chang Lin,[f] Yusuke Yamauchi,[h,i] Brian Yuliarto,[j,k] Watchareeya Kaveevivitchai,[l] Mohammad Abu Haija,[a,b]* and Ahmed F. M. EL-Mahdy[d]*

[a] *Department of Chemistry, Khalifa University of Science and Technology, Abu Dhabi, P.O. Box 127788, United Arab Emirates*

[b] *Center for Catalysis and Separations (CeCaS), Khalifa University of Science and Technology, Abu Dhabi, P.O. Box 127788, United Arab Emirates*

[c] *Chemistry Department, Faculty of Science, Tanta University, Tanta 31527, Egypt*

[d] *Department of Materials and Optoelectronic Science, National Sun Yat-Sen University, Kaohsiung, 80424, Taiwan*

[e] *Physics Department, Faculty of Science, Tanta University, Al-Geish St., Tanta 31527, Egypt*

[f] *Department of Applied Chemistry, National Yang Ming Chiao Tung University, Hsinchu 30010, Taiwan*

[g] *Institute of Medical Science and Technology, National Sun Yat-sen University, Kaohsiung 804201, Taiwan*

[h] *Australian Institute for Bioengineering and Nanotechnology, The University of Queensland, Brisbane, QLD 4072, Australia*

[i] *Department of Materials Process Engineering, Graduate School of Engineering, Nagoya University, Nagoya, 464-8603, Japan*

[j] *Advanced Functional Materials Laboratory, Engineering Physics Department, Faculty of Industrial Technology, Institut Teknologi Bandung, Bandung 40132, Indonesia*

[k] *Research Center for Nanoscience and Nanotechnology (RCNN), Institut Teknologi Bandung, Bandung 40132, Indonesia*

[l] *Department of Chemical Engineering, Hierarchical Green-Energy Materials (Hi-GEM) Research Center, Academy of Innovative Semiconductor and Sustainable Manufacturing, National Cheng Kung University, Tainan City 70101, Taiwan*

Corresponding Authors E-mails: mohammad.abuhaija@ku.ac.ae (M. Abu Haija); ahmedelmahdy@mail.nsysu.edu.tw (A. F. M. EL-Mahdy)

1. Chemicals

Starting chemicals, reagents, and solvents are of analytical grade and were purchased from commercial providers. All chemicals were used for chemical reaction synthesis without any further purifications. 2,3-dihydroxynaphthalene, 2,6-dihydroxynaphthalene, 4-bromoaniline, tetrakis(triphenylphosphine)palladium(0), potassium carbonate, formamidine, [1,1′-bis(diphenylphosphino)ferrocene]dichloropalladium(II), acetic anhydride, HCl, dioxane, chloroform, triflic acid, pyrene, bromine, acetate, bis(pinacolato)diboron, potassium acetate, *n*-butanol and *o*-dichlorobenzene, acetic acid, *N*,*N′*-dimethyl formamide (DMF), tetrahydrofuran (THF) and acetone were purchased and utilized as received.

2. Synthetic Procedures

2.1 Synthesis of 2,3-dihydroxynaphthalene-1,4-dicarbaldehyde (2,3-NADC)

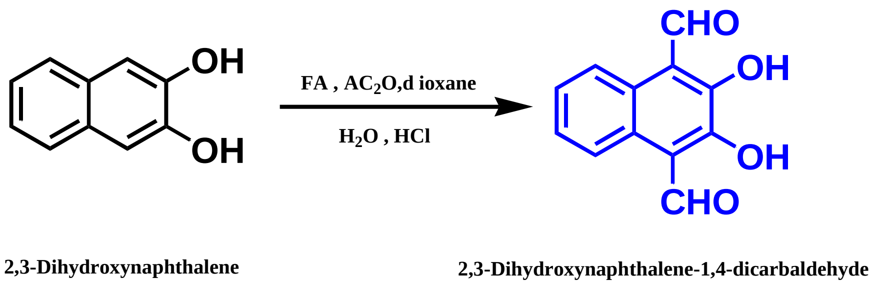

Scheme S1. Synthesis of 2,3-dihydroxynaphthalene-1,4-dicarbaldehyde.

Formamidine acetate (1.56 g, 14.98 mmol) and dioxane (30 mL) were added to a 100-mL two-neck round-bottom flask and heated under reflux. Acetic anhydride (3 mL) was added when the target temperature reached 95 °C, and then the mixture was stirred for 30 min. Subsequently, 2,3-dihydroxynaphthalene (300 mg, 1.87 mmol) was added once all formamidine acetate was dissolved, and the solution was kept for two days. After cooling, dioxane was evaporated at 50 °C. Deionized water (45 mL) was added, and the mixture was heated at 65 °C for 2 h. Then, HCl (1 M, 40 mL) was added and kept under heating at 65 °C for 18 h. The solid precipitate was filtered with hexane. Finally, 2,3-dihydroxynaphthalene-1,4-dicarbaldehyde was obtained after purification using a purification column (Scheme S1). ^{1}H NMR (DMSO-d_6, 25 °C, 500 MHz) δ (ppm): 10.83 (s, 2H), 8.76 (*d*, *J* =, 2H), 7.51 (*d*, *J* = Hz, 2H) (Figure S1). ^{13}C NMR (DMSO-d_6, 25 °C, 125 MHz) δ (ppm): 194.15, 154.29, 126.30, 125.38, 122.32, 117.05 (Figure S2).

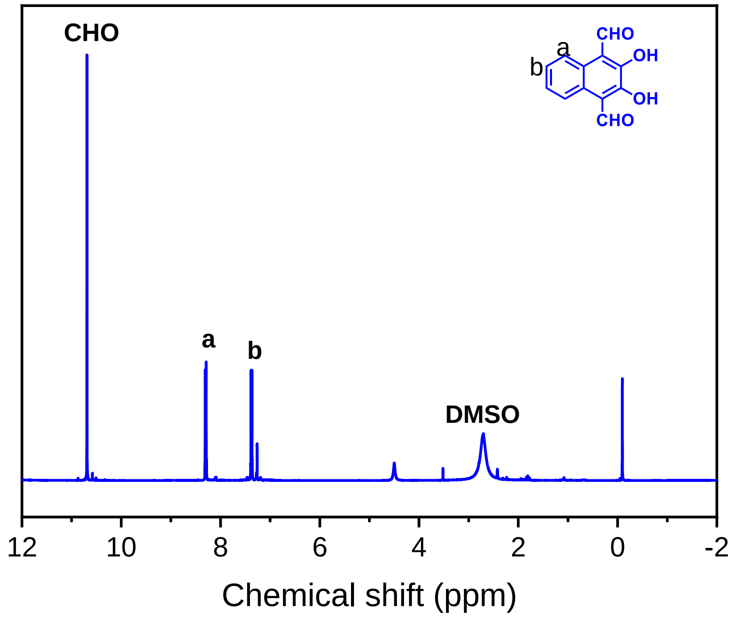

Figure S1. ^{1}H-NMR of 2,3-dihydroxynaphthalene-1,4-dicarbaldehyde.

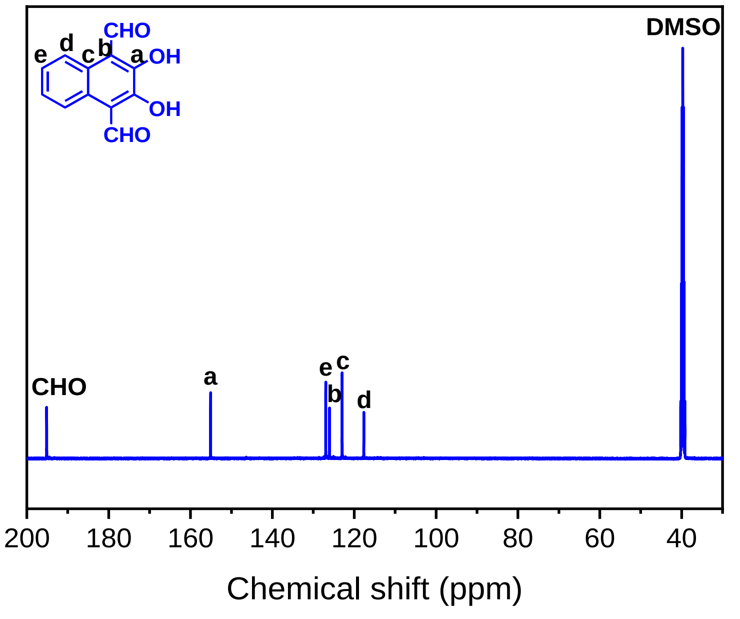

Figure S2. ^{13}C-NMR of 2,3-dihydroxynaphthalene-1,4-dicarbaldehyde.

2.2 Synthesis of 2,6-dihydroxynaphthalene-1,5-dicarbaldehyde (2,6-NADC)

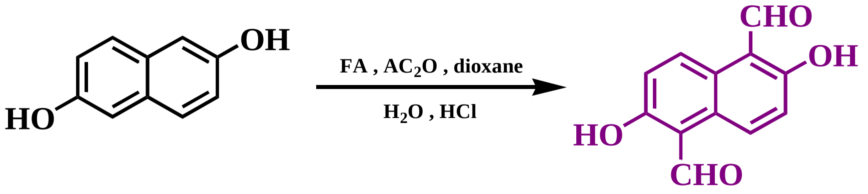

2,6-Dihydroxynaphthalene **2,6-Dihydroxynaphthalene-1,5-dicarbaldehyde**

Scheme S2. Synthesis of 2,6-dihydroxynaphthalene-1,5-dicarbaldehyde.

Formamidine acetate (1.56 g, 14.98 mmol) and dioxane (45 mL) were heated under reflux in a 100-mL two-neck round-bottom flask. Acetic anhydride (3 mL) was added when the target temperature reached 95 °C and stirred for thirty minutes. Subsequently, 2,6-dihydroxynaphthalene (300 mg, 1.87 mmol) was added once all formamidine acetate was dissolved and kept for two days. After cooling for minutes, dioxane was evaporated at 50 °C. Then, water (45 mL) was added, and the mixture was heated up to 65 °C for 2 h. Then, HCl (1 M, 45 mL) was added, and the temperature was maintained at 65 °C for 18 h. The powder was filtered with hexane, and 2,6-dihydroxynaphthalene-1,5-dicarbaldehyde was obtained after purification using a purification column (Scheme S2). ^{1}H NMR (DMSO-d_6, 25 °C, 500 MHz) δ (ppm): 10.75 (s, 2H), 9.14 (*d*, *J* = Hz, 2H), 7.32 (*d*, *J* = Hz, 2H) (Figure S3). ^{13}C NMR (DMSO-d_6, 25 °C, 125 MHz) δ (ppm): 192.74, 161.56, 132.39, 125.86, 121.35, 113.34 (Figure S4).

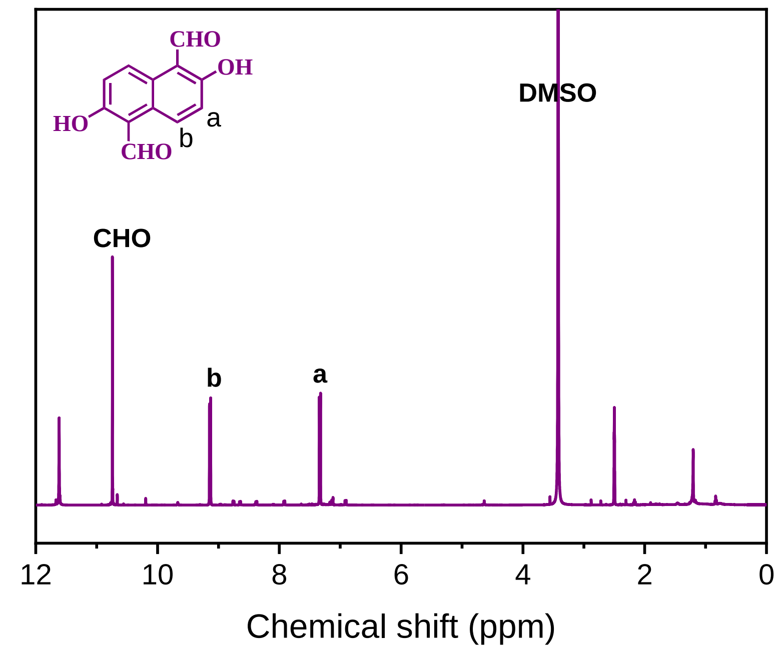

Figure S3. ^{1}H-NMR of 2,6-dihydroxynaphthalene-1,5-dicarbaldehyde.

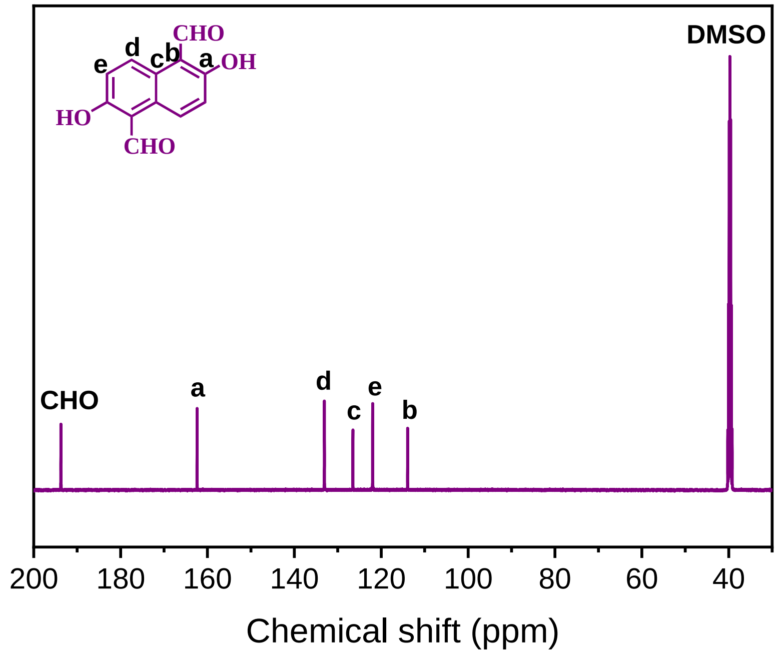

Figure S4. ^{13}C-NMR of 2,3-dihydroxynaphthalene-1,4-dicarbaldehyde.

2.3 Synthesis of 1,3,6,8-tetrakis(4-aminophenyl)pyrene (PyTA-4NH$_2$)

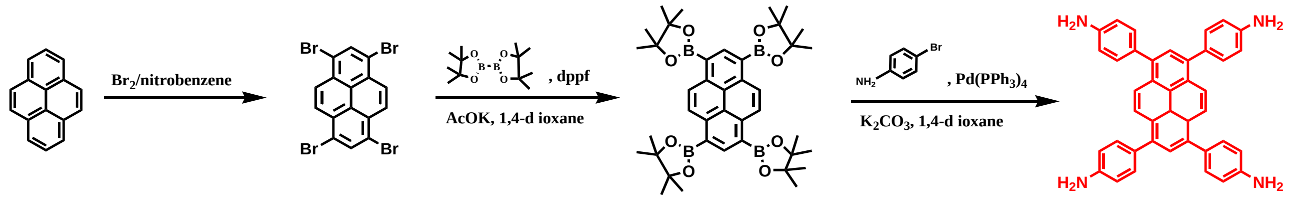

pyrene Pyrene-4Br 1,3,6,8-tetrakis(4,4,5,5-tetramethyl-1,3,2-dioxaborolan-2-yl)pyrene 1,3,6,8-tetrakis(4-aminophenyl)pyrene

Scheme S3. Synthesis of 1,3,6,8-tetrakis(4-aminophenyl)pyrene (PyTA-4NH$_2$).

2.3.1 1,3,6,8-tetrabromopyrene (Pyrene-4Br): A 250 mL round-bottom flask was filled with pyrene (2.5 g, 12 mmol) and nitrobenzene (100 mL), refluxing at 120 °C for 15 h. Subsequently, bromine (2.8 mL, 54.5 mmol) was added dropwise. The solid precipitate was filtered and washed with ethanol.

2.3.2 1,3,6,8-tetrakis(4-aminophenyl)pyrene (PyTA-4NH$_2$): Pyrene-4Br (1.0 g, 1.9 mmol), bis(pinacolato)diboron (2.99 g, 11.78 mmol), [1,1′-bis(diphenylphosphino)ferrocene]dichloro palladium(II) (120.5 mg, 0.0165 mmol), and potassium acetate (1.17 g, 11.69 mmol) were filled in a round-bottom flask and evacuated for twenty minutes. Subsequently, dioxane (20 mL) was added to the mixture and refluxed for 48 h under N$_2$. The mixture was cooled to room temperature and poured into ice water. Water was added to filter the yellow precipitate, and then THF/hexane was used as an eluent in flash column chromatography to purify. After that, 1,3,6,8-tetrakis(4,4,5,5-tetramethyl-1,3,2-dioxaborolan-2-yl)pyrene (TTDBPy) was obtained after recrystallization with methanol. Subsequently, TTDBPy (0.5 g, 0.75 mmol), 4-bromoaniline (0.98 g, 5.65 mmol), tetrakis(triphenylphosphine)palladium(0) (40.44 mg, 0.035 mmol), and potassium carbonate (0.975 g, 7.05 mmol) were filled in a round-bottom flask and evacuated under high pressure for 20 min. Then, a mixture of dioxane (20 mL) and water (3.5 mL) was added and heated at 100 °C for 48 h under N$_2$. Finally, the mixture was cooled down to room temperature and poured into ice water. The yellow-greenish precipitate was filtered with water, methanol, and dichloromethane. The isolated solid of 4,4',4'',4'''-pyrene-1,3,6,8-tetrayl)tetraaniline (PyTA-4NH$_2$) was obtained without purification (Scheme S3). ^{1}H NMR (500 MHz, 25 °C, DMSO-d_6) δ (ppm): 8.12 (s, 4H), 7.79 (s, 2H), 7.35 (*d*, *J* = 12 Hz, 8H), 6.78 (*d*, *J* = 12 Hz, 8H), 5.30 (s, br., 8H, 4NH2) (Figure S5). ^{13}C S6 NMR (125 MHz, DMSO) δ (ppm): 148.21, 137.14, 131.06, 129.04, 127.60, 126.72, 126.13, 124.43, 113.96 (Figure S6).

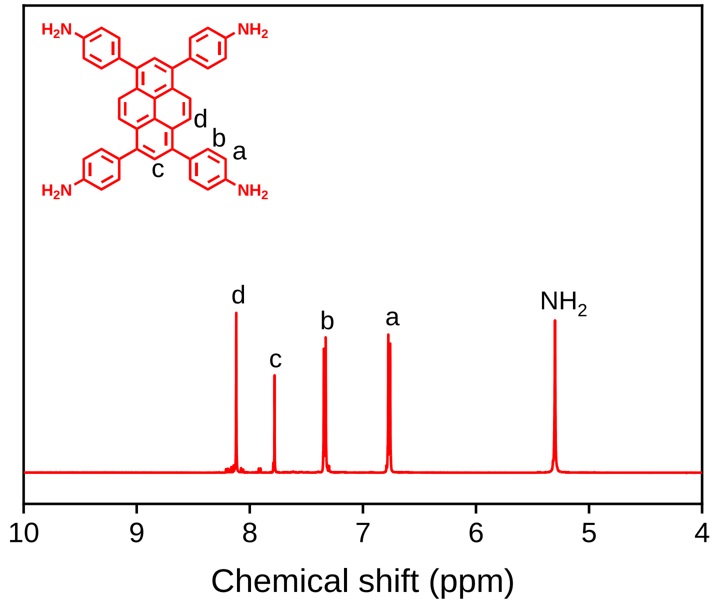

Figure S5. ^{1}H-NMR of 1,3,6,8-tetrakis(4-aminophenyl)pyrene (PyTA-4NH2).

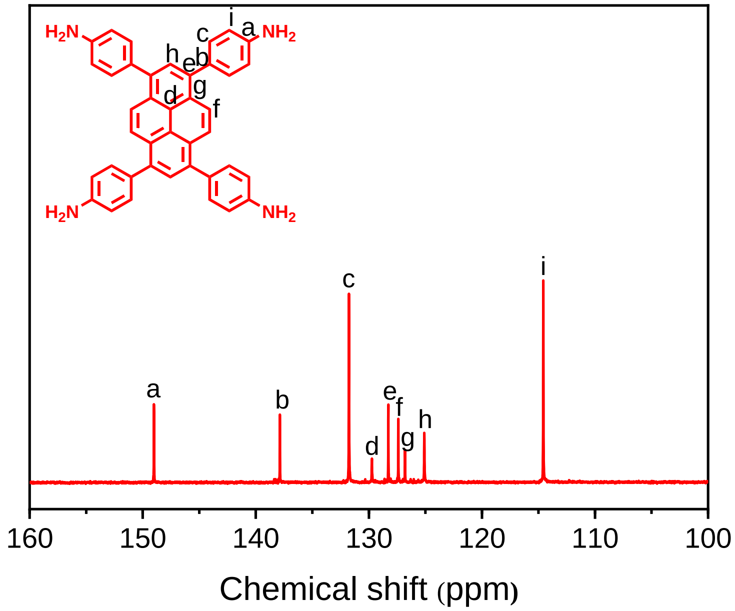

Figure S6. ^{13}C-NMR of 1,3,6,8-tetrakis(4-aminophenyl)pyrene (PyTA-4NH2).

2.4 Synthesis of PyTA-2,3-NA(OH)$_2$ HO-COF

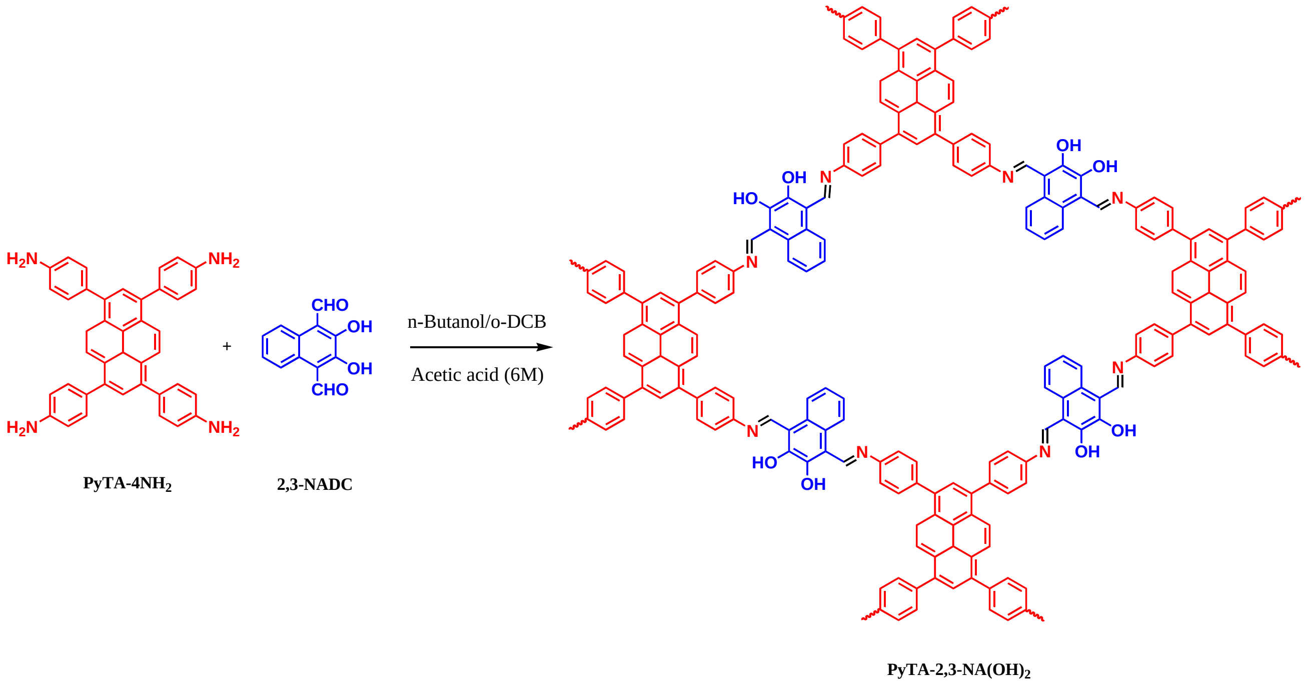

Scheme S4. Synthesis of PyTA-2,3-NA(OH)$_2$ HO-COF.

Three freeze/pump/thaw cycles were used to degas the mixture solution of PyTA-4NH$_2$ (70 mg, 0.124 mmol) and 2,3-NADC (53.41 mg, 0.247 mmol) in *n*-butanol (3.5 mL) and *o*-dichlorobenzene (3.5 mL) with acetic acid (6 M, 0.7 mL) in a 25-mL Schlenk storage tube. The tube was sealed with a flame and heated for three days at 120 °C. The tube was opened after cooling to room temperature, and the precipitate was filtered and washed (once with DMF, three times each with THF and acetone). The PyTA-2,3-NA(OH)$_2$ COF was obtained as an orange powder by drying the solid under vacuum at 120 °C overnight.

2.5 Synthesis of PyTA-2,6-NA(OH)$_2$ HO-COF

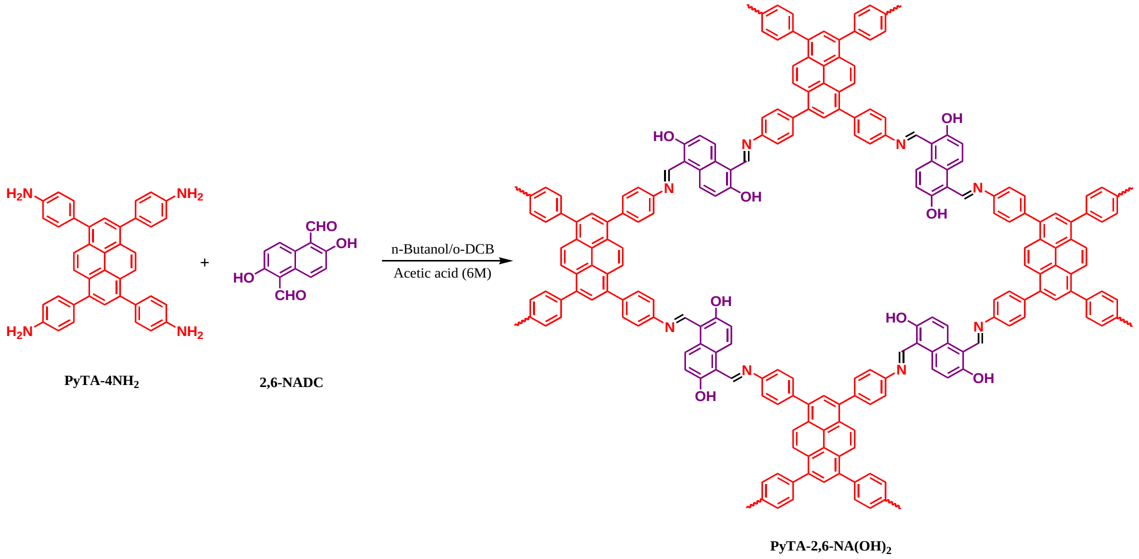

Scheme S5. Synthesis of PyTA-2,6-NA(OH)$_2$ HO-COF.

Three freeze/pump/thaw cycles were used to degas the mixture solution of PyTA-4NH$_2$ (70 mg, 0.124 mmol) and 2,6-NADC (53.41 mg, 0.247 mmol) in *n*-butanol (3.5 mL) and *o*-dichlorobenzene (3.5 mL) with acetic acid (6 M, 0.7 mL) in a 25-mL Schlenk storage tube. The tube was sealed with a flame and heated for three days at 120 °C. Then, the tube was opened after cooling to room temperature, and the precipitate was filtered and washed (once with DMF, three times each with THF and acetone). The PyTA-2,6-NA(OH)$_2$ HO-COF was obtained as a purple powder by drying the solid under vacuum at 120 °C overnight.

3. Characterization of HO-COFs

3.1 PyTA-2,3-NA(OH)$_2$ HO-COF

As shown in Scheme S4, the PyTA-2,3-NA(OH)$_2$ COF was synthesized using the solvothermal method carried out at 120 °C for three days with PyTA-4NH$_2$ and 2,3-NADC in *n*-butanol and *o*-dichlorobenzene with 6 M CH_3COOH as a catalyst. FTIR spectrum of PyTA-2,3-NA(OH)$_2$ HO-COF did not exhibit the aldehydic C-H band and the aldehydic C=O band of 2,3-NADC. It also reflected a C=N band at 1625 cm^{-1} (Figure S7).

3.2 PyTA-2,6-NA(OH)$_2$ HO-COF

As shown in Scheme S5, the PyTA-2,6-NA(OH)$_2$ COF was synthesized using the solvothermal method carried out at 120 °C for three days with PyTA-4NH$_2$ and 2,6-NADC in *n*-butanol and *o*-dichlorobenzene with 6 M CH_3COOH as a catalyst. FTIR spectrum of PyTA-2,6-NA(OH)$_2$ HO-COF did not exhibit the aldehydic C-H band and aldehydic C=O band of 2,6-NADC. It also reflected a C=N band at 1620 cm^{-1} (Figure S8).

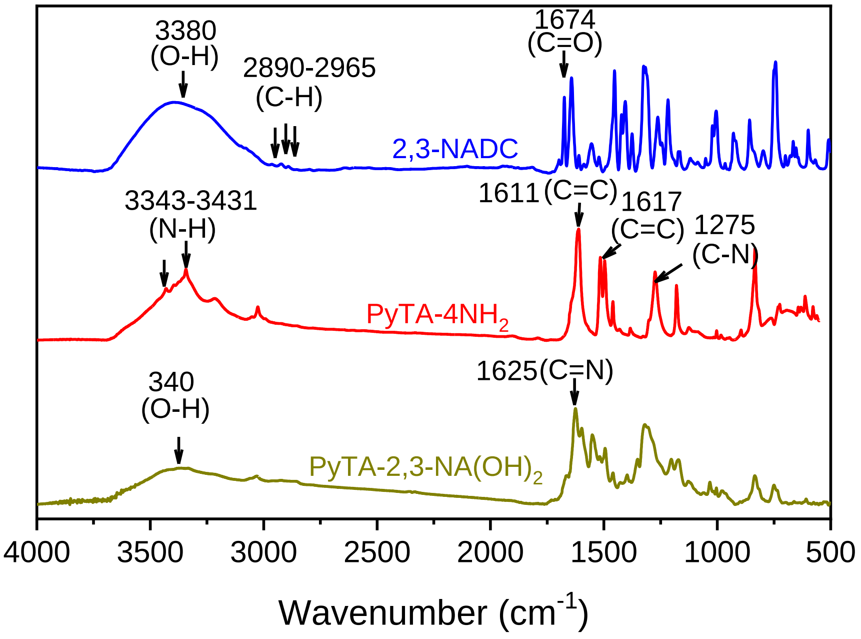

Figure S7. FTIR spectrum of 2,3-NADC (blue line), PyTA-4NH$_2$ (red line), and PyTA-2,3-NA(OH)$_2$ HO-COF (dark yellow line).

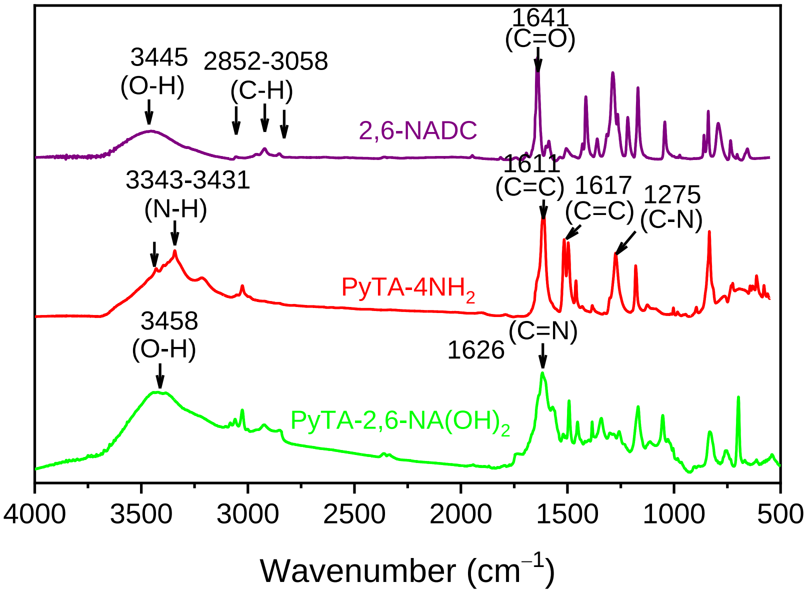

Figure S8. FTIR spectrum of 2,6-NADC (purple line), PyTA-4NH_2 (red line), and PyTA-2,6-NA$(OH)_2$ HO-COFs (green line).

4. Materials Characterizations

INOVA 500 (INOVA 500MHz NMR, INOVA 500 instrument) was used for recording ^{1}H and ^{13}C NMR portfolios through DMSO-d_6 and $CDCl_3$ as exterior solvents, and chemical shifts were detected in parts per million (ppm). Bruker advance III HD (in National Cheng Kung University and a Bruker magic-angle-spinning (MAS) probe, running 32,000 scans). A Bruker Advance 400 NMR spectrometer and Bruker magic angle spinning (MAS) probe were employed to obtain the solid state ^{13}C NMR (Solid state nuclear magnetic resonance SSNMR) portfolios. ^{13}C NMR spectral data were acquired through cross-polarization with MAS (CPMAS) at 75.5 MHz. Fourier-transform infrared spectrophotometer (FTIR, Bruker Tensor 27) was used to record FTIR spectra of the synthesized samples. A transmission electron microscope (TEM, JEOL-2100) equipped with a field-emission microscope (JEOL, Tokyo, Japan) operating at a high voltage of 200 kV and a field emission scanning electron microscope (FE-SEM, JEOL JSM-7610F) operating at an accelerating voltage of 5 kV was used to investigate the morphological structure of the obtained COF materials. A TA Q-50 Thermogravimetric Analyzer was used to perform thermogravimetric (TG) analysis in N_2 heating from room temperature to 800 °C at a rate of 20 °C min^{-1}. Powder X-ray diffraction (PXRD) was performed using a Siemens D5000 diffractometer (40 kV/30 mA) with Cu Kα radiation source (λ = 1.540 Å) at a scan rate of 2° min^{-1}.

Material Studio software was utilized for COF structural simulations based on unit cell dimensions, which were determined manually from the observed XRD peak positions using the coordinates. The surface area and porosity analyzer BelSorp Max equipment was utilized to measure the Ar adsorption-desorption isotherms of the HO-COFs samples in liquid nitrogen at 87 K. The specific surface areas and pore structural properties of the obtained COF materials were analyzed by applying the multipoint Brunauer-Emmett-Teller (BET), *t*-plot, and quenched solid density functional theory (QSDFT) methods, respectively. The surface areas of the polymer CMP materials were calculated based on the BET model by using the data of adsorption branches in the relative pressure (P/P_0) range of 0.05-0.2. Before the measurements, the samples were degassed under vacuum at 100 °C for 8 h. UV-vis diffuse reflectance spectra (UV-vis DRS) were recorded at 25 °C using a Jasco V-570 spectrometer (Japan). The chemical states and elemental compositions were evaluated from X-ray photoelectron spectroscopy (XPS) spectra using a British VG Scientific ESCALAB 250 system with an Al Kα X-ray radiation source at 1486.6 eV. All the binding energies were calibrated *by referencing* C1s binding energy (285.0 eV).

Table S1. Fractional atomic coordinates for the unit cell of PyTA-2,3-NA(OH)$_2$ HO-COF with A–A stacking.

Sample name: PyTA-2,3-NA(OH)$_2$ HO-COF							
Space group: P 1							
a = 35.5 Å, b =32.4 Å, c = 3.6 Å, α = β = γ = 90°							
Rwp = 17.12%, Rp = 12.87%							
Atom	x/a	y/b	z/c	Atom	x/a	y/b	z/c
C1	0.48936	0.99515	-0.25285	N111	0.67246	0.19028	-0.75701
C2	0.52058	0.96810	-0.29654	O112	0.80904	0.22317	-0.37574
C3	0.55450	0.98633	-0.39223	O113	0.74170	0.18383	-0.55456
C4	0.42316	1.00583	-0.20274	N114	0.35839	0.23786	0.17235
C5	0.45274	0.97803	-0.20798	C115	0.32500	0.24043	0.02321
C6	0.44705	0.93408	-0.20182	C116	0.29610	0.26917	0.15949
C7	0.47869	0.90926	-0.27148	C117	0.30045	0.31246	0.14490
C8	0.51597	0.92413	-0.27999	C118	0.26868	0.33835	0.21158
C9	0.54735	0.89197	-0.21152	C119	0.23334	0.32033	0.30743
C10	0.41088	0.91130	-0.09031	C120	0.23038	0.27716	0.35008
C11	0.53829	0.85559	-0.00662	C121	0.26124	0.25172	0.25478
C12	0.56474	0.82431	0.06115	C122	0.33531	0.33054	0.04591
C13	0.60212	0.82997	-0.03932	C123	0.33887	0.37320	0.01323
C14	0.61230	0.86588	-0.22858	C12	0.30784	0.39844	0.07080
C15	0.58546	0.89490	-0.33333	C125	0.27303	0.38143	0.16572
C16	0.37357	0.92419	-0.17645	C126	0.19898	0.34600	0.35365
C17	0.34221	0.90203	-0.05943	O127	0.19733	0.26014	0.50064
C18	0.34617	0.86410	0.12111	O128	0.25804	0.20848	0.24569

C19	0.38214	0.84931	0.19579	N129	0.16637	0.33234	0.24723
C20	0.41378	0.87254	0.09622	N130	0.14772	0.71373	0.39007
N21	0.31429	0.83995	0.23470	C131	0.15030	0.74956	0.54739
C22	0.95326	0.60041	0.33856	C132	0.18537	0.77335	0.50226
C23	0.95639	0.55861	0.21900	C133	0.22105	0.75373	0.47638
C24	0.99101	0.53721	0.25428	C134	0.25251	0.77605	0.33610
C25	0.99371	0.49435	0.15094	C135	0.24867	0.81896	0.26190
C26	0.92572	0.53709	0.07212	C136	0.21420	0.83895	0.32983
C27	0.92802	0.49611	-0.02884	C137	0.18219	0.81583	0.43969
C28	0.96197	0.47313	0.00211	C138	0.22616	0.71206	0.58709
C29	0.96531	0.42981	-0.09187	C139	0.26038	0.69198	0.53253
C30	1.00034	0.41045	-0.04573	C140	0.29037	0.71307	0.37227
C31	0.91769	0.62561	0.31212	C141	0.28649	0.75444	0.27011
C32	0.93333	0.40288	-0.23038	O142	0.14627	0.83400	0.46972
C33	0.91891	0.66618	0.16838	O143	0.21191	0.88211	0.28928
C34	0.88611	0.69013	0.14554	C144	0.27979	0.84506	0.12303
C35	0.85155	0.67446	0.26868	H145	0.57614	0.96947	-0.51126
C36	0.85012	0.63420	0.41317	H146	0.39495	0.99694	-0.22069
C37	0.88285	0.61070	0.44181	H147	0.47483	0.87660	-0.31056
C38	0.90499	0.42011	-0.44682	H148	0.51091	0.85126	0.11866
C39	0.87326	0.39864	-0.55582	H149	0.55674	0.79703	0.21782
C40	0.86984	0.35700	-0.47028	H150	0.64104	0.87057	-0.31587
C41	0.89882	0.33713	-0.27277	H151	0.59678	0.91597	-0.53510
C42	0.93031	0.36004	-0.14957	H152	0.36605	0.94906	-0.35452

C43	0.83709	0.33573	-0.62722	H153	0.31490	0.91438	-0.12368
C44	0.46850	0.11061	-0.21443	H154	0.38531	0.81973	0.33542
C45	0.46321	0.06662	-0.23327	H155	0.44064	0.85992	0.17250
C46	0.49465	0.03965	-0.26803	H156	0.89869	0.55082	0.04064
C47	0.53096	0.05650	-0.34303	H157	0.90057	0.48333	-0.10192
C48	0.53619	0.10040	-0.37587	H158	1.00367	0.37911	-0.14179
C49	0.50482	0.12558	-0.29305	H159	0.94506	0.67915	0.06595
C50	0.55974	0.02819	-0.41136	H160	0.88729	0.72099	0.02892
C51	0.42800	0.04787	-0.23410	H161	0.82390	0.62047	0.50364
C52	0.57228	0.12267	-0.50169	H162	0.88024	0.58050	0.55780
C53	0.43877	0.14279	-0.10119	H163	0.90843	0.45072	-0.53944
C54	0.60905	0.10904	-0.41224	H164	0.85215	0.41434	-0.72255
C55	0.64100	0.13024	-0.52405	H165	0.89656	0.30451	-0.20961
C56	0.63815	0.16838	-0.69537	H166	0.95113	0.34462	0.02167
C57	0.60258	0.18452	-0.77807	H167	0.82751	0.34740	-0.88651
C58	0.57007	0.16164	-0.68740	H168	0.50868	0.15842	-0.29430
C59	0.44943	0.17956	0.08870	H169	0.58681	0.03588	-0.50059
C60	0.42315	0.21088	0.17155	H170	0.40287	0.06281	-0.31127
C61	0.38524	0.20615	0.07812	H171	0.61547	0.08426	-0.23403
C62	0.37410	0.16934	-0.09387	H172	0.66872	0.11876	-0.44984
C63	0.40043	0.13986	-0.19837	H173	0.60011	0.21404	-0.92072
C64	0.02289	0.55830	0.40261	H174	0.54325	0.17482	-0.76433
C65	0.02132	0.60183	0.46965	H175	0.47797	0.18443	0.17972
C66	-0.01442	0.62036	0.47095	H176	0.43231	0.23860	0.31512

C67	0.02787	0.47268	0.21188	H177	0.34508	0.16314	-0.16245
C68	0.05670	0.49330	0.39918	H178	0.38940	0.11764	-0.38319
C69	0.05373	0.53403	0.50361	H179	-0.01707	0.65192	0.56796
C70	0.03160	0.43051	0.10777	H180	0.08119	0.47762	0.49490
C71	0.05484	0.63114	0.47015	H181	0.07297	0.54260	0.71601
C72	0.06724	0.40568	0.15646	H182	0.09879	0.59420	0.75429
C73	0.09130	0.62116	0.60482	H183	0.14892	0.63974	0.66782
C74	0.12124	0.64837	0.56765	H184	0.07604	0.73001	0.17946
C75	0.11589	0.68748	0.42246	H185	0.02410	0.68111	0.18627
C76	0.08016	0.69964	0.30152	H186	0.03885	0.34994	0.34435
C77	0.05026	0.67148	0.31460	H187	0.09657	0.30838	0.39392
C78	0.06549	0.36405	0.27406	H188	0.16252	0.41178	0.03506
C79	0.09819	0.34022	0.30573	H189	0.10664	0.45306	-0.02979
C80	0.13336	0.35747	0.22909	H190	0.67149	0.84159	0.18137
C81	0.13554	0.39852	0.10950	H191	0.62625	0.74338	0.46244
C82	0.10302	0.42199	0.06670	H192	0.61423	0.67069	0.56228
N83	0.62986	0.79968	0.06191	H193	0.66587	0.61997	0.53440
C84	0.66379	0.80937	0.16282	H194	0.73008	0.64228	0.41631
C85	0.69365	0.77831	0.24099	H195	0.78316	0.66929	0.59757
C86	0.68622	0.73662	0.34338	H196	0.74902	0.83625	-0.14786
C87	0.71670	0.70798	0.36518	H197	0.80128	0.80852	0.16643
C88	0.75440	0.72212	0.32991	H198	0.77518	0.37528	-0.67999
C89	0.76145	0.76408	0.25601	H199	0.72308	0.41139	-0.93984
C90	0.73108	0.79161	0.20075	H200	0.66708	0.37418	-1.15663

C91	0.64961	0.72252	0.43084	H201	0.66363	0.29928	-1.11161
C92	0.64255	0.68085	0.49838	H202	0.65196	0.24923	-0.72086
C93	0.67177	0.65237	0.48556	H203	0.83294	0.23910	-0.32081
C94	0.70852	0.66569	0.42155	H204	0.76476	0.17076	-0.43439
C95	0.78659	0.69399	0.39805	H205	0.31572	0.21875	-0.18917
N96	0.81864	0.69974	0.23040	H206	0.35973	0.31193	-0.01239
O97	0.73832	0.83315	0.11513	H207	0.36568	0.38682	-0.06357
O98	0.79874	0.77867	0.24907	H208	0.31084	0.43155	0.03884
N99	0.81748	0.30856	-0.44951	H209	0.24991	0.40264	0.19731
C100	0.78319	0.28974	-0.58408	H210	0.20139	0.37714	0.45941
C101	0.75212	0.31279	-0.73293	H211	0.20023	0.23207	0.61743
C102	0.71829	0.29176	-0.83305	H212	0.23160	0.19833	0.19291
C103	0.71429	0.24903	-0.75854	H213	0.12673	0.76272	0.70261
C104	0.74555	0.22636	-0.62436	H214	0.20427	0.69504	0.72592
C105	0.77951	0.24670	-0.53344	H215	0.26372	0.65993	0.61362
C106	0.75257	0.35635	-0.77071	H216	0.31664	0.69716	0.32159
C107	0.72206	0.37809	-0.91743	H217	0.31014	0.76834	0.12732
C108	0.69018	0.35709	-1.03320	H218	0.14400	0.86217	0.34984
C109	0.68826	0.31433	-0.99569	H219	0.19610	0.89488	0.49520
C110	0.67631	0.22987	-0.76452	H220	0.27235	0.86941	-0.06964

Table S2. Fractional atomic coordinates for the unit cell of PyTA-2,6-NA(OH)$_2$ HO-COF with A–A stacking.

Sample name: PyTA-2,6-NA(OH)$_2$ HO-COF							
Space group: P 1							
a = 34.8 Å, b =32.4 Å, c = 3.8 Å, α = β = γ = 90°							
Rwp = 12.66%, Rp = 9.36%							
Atom	x/a	y/b	z/c	Atom	x/a	y/b	z/c
C1	0.42830	0.96395	0.97153	C111	0.69701	0.23484	0.59838
C2	0.46328	0.94450	1.03470	C112	0.66106	0.21208	0.64486
C3	0.49783	0.96622	0.98181	O113	0.83797	0.18285	1.14767
C4	0.53383	0.94746	1.04218	O114	0.65936	0.28311	0.28479
C5	0.56733	0.96980	0.98569	N115	0.65983	0.17376	0.58474
C6	0.46486	0.90385	1.14218	C116	0.30140	0.16684	0.29815
C7	0.50067	0.88632	1.20329	C117	0.26985	0.19693	0.28190
C8	0.53514	0.90678	1.14995	C118	0.27242	0.23683	0.41313
C9	0.57143	0.88343	1.19637	C119	0.23929	0.26207	0.41266
C10	0.43059	0.87717	1.17597	C120	0.20556	0.24808	0.25812
C11	0.39894	0.88766	1.37471	C121	0.20399	0.21017	0.11414
C12	0.36815	0.86125	1.41467	C122	0.23561	0.18454	0.12593
C13	0.36873	0.82325	1.26739	C123	0.30723	0.25301	0.53090
C14	0.40044	0.81231	1.06950	C124	0.30872	0.29132	0.66840
C15	0.43090	0.83905	1.02427	C125	0.27628	0.31526	0.68716
C16	0.57533	0.84514	1.05154	C126	0.24114	0.30131	0.55459
C17	0.60874	0.82254	1.10041	C127	0.20750	0.32834	0.55761
C18	0.63902	0.83762	1.29256	O128	0.23330	0.14642	-0.01921

C19	0.63514	0.87585	1.43808	O129	0.28059	0.35247	0.84560
C20	0.60149	0.89792	1.39529	N130	0.17543	0.31714	0.69525
N21	0.67286	0.81331	1.33180	N131	0.15658	0.67613	1.28125
N22	0.33681	0.79699	1.32939	C132	0.15603	0.71511	1.27485
C23	0.02919	0.52946	1.18452	C133	0.19149	0.73879	1.21836
C24	0.02690	0.56988	1.29525	C134	0.22875	0.72656	1.32718
C25	-0.00898	0.58607	1.37343	C135	0.26156	0.74952	1.23221
C26	0.03359	0.44850	0.95488	C136	0.25625	0.78429	1.03709
C27	0.06659	0.47199	0.99985	C137	0.21971	0.79669	0.94341
C28	0.06439	0.51154	1.10384	C138	0.18746	0.77430	1.03128
C29	0.03569	0.40780	0.84812	C139	0.23426	0.69254	1.53136
C30	0.06035	0.59784	1.30350	C140	0.27097	0.67995	1.61916
C31	0.07237	0.38512	0.80308	C141	0.30321	0.70111	1.51786
C32	0.09403	0.58878	1.48089	C142	0.29895	0.73671	1.32905
C33	0.12550	0.61476	1.47069	O143	0.15113	0.78618	0.91997
C34	0.12318	0.65120	1.29966	O144	0.33878	0.68642	1.62685
C35	0.08907	0.66134	1.13355	H145	0.40115	0.94907	1.00006
C36	0.05803	0.63478	1.13430	H146	0.59554	0.95736	1.01812
C37	0.07694	0.34738	0.95630	H147	0.50175	0.85563	1.29298
C38	0.11081	0.32542	0.91461	H148	0.39811	0.91587	1.50470
C39	0.14103	0.34072	0.72362	H149	0.34413	0.86976	1.57069
C40	0.13625	0.37795	0.56278	H150	0.40184	0.78370	0.94406
C41	0.10208	0.39941	0.59856	H151	0.45474	0.83013	0.86716
C42	0.95687	0.56469	1.32708	H152	0.55272	0.83305	0.89559

C43	0.95890	0.52413	1.21717	H153	0.61137	0.79333	0.98505
C44	0.99516	0.50649	1.14597	H154	0.65746	0.88897	1.58895
C45	0.92589	0.50084	1.16804	H155	0.59912	0.92648	1.51759
C46	0.92807	0.46129	1.06337	H156	-0.01037	0.61674	1.46285
C47	0.96329	0.44316	0.98634	H157	0.09501	0.46039	0.96102
C48	0.99731	0.46609	1.02633	H158	0.09114	0.52748	1.11984
C49	0.96563	0.40266	0.87655	H159	0.09629	0.56146	1.62357
C50	1.00162	0.38631	0.80322	H160	0.15167	0.60697	1.60076
C51	0.92033	0.58783	1.36662	H161	0.08711	0.68880	0.99033
C52	0.93202	0.37498	0.85756	H162	0.03250	0.64250	0.99389
C53	0.91690	0.62557	1.21158	H163	0.05459	0.33529	1.11501
C54	0.88409	0.64901	1.25718	H164	0.11404	0.29672	1.03767
C55	0.85384	0.63540	1.45599	H165	0.15795	0.39016	0.39988
C56	0.85668	0.59749	1.60773	H166	0.09886	0.42700	0.46260
C57	0.88996	0.57446	1.56941	H167	0.89753	0.51261	1.20408
C58	0.89882	0.38486	0.67613	H168	0.90131	0.44543	1.04585
C59	0.86734	0.35890	0.67060	H169	1.00317	0.35563	0.71412
C60	0.86936	0.32160	0.82795	H170	0.93954	0.63675	1.05127
C61	0.90292	0.31061	0.99780	H171	0.88198	0.67796	1.13564
C62	0.93386	0.33723	1.01518	H172	0.83383	0.58571	1.76110
N63	0.83632	0.29619	0.82745	H173	0.89197	0.54675	1.70584
C64	0.56593	0.00921	0.87756	H174	0.89695	0.41279	0.54185
C65	0.53099	0.02833	0.80905	H175	0.84160	0.36729	0.53688
C66	0.49643	0.00661	0.86169	H176	0.90447	0.28221	1.12771

C67	0.46046	0.02547	0.80269	H177	0.95895	0.32880	1.15850
C68	0.42692	0.00345	0.86368	H178	0.59307	0.02417	0.85057
C69	0.52950	0.06875	0.69703	H179	0.39881	0.01622	0.83088
C70	0.49380	0.08609	0.63104	H180	0.49285	0.11670	0.53898
C71	0.45929	0.06596	0.69155	H181	0.59701	0.05726	0.34484
C72	0.42342	0.09024	0.66038	H182	0.65216	0.10254	0.31055
C73	0.56369	0.09559	0.67027	H183	0.59319	0.18763	0.93551
C74	0.59596	0.08522	0.47879	H184	0.53881	0.14204	0.98118
C75	0.62738	0.11111	0.45690	H185	0.44498	0.13848	0.97078
C76	0.62664	0.14854	0.61335	H186	0.38885	0.18196	0.89060
C77	0.59419	0.15956	0.80074	H187	0.33650	0.09151	0.27336
C78	0.56310	0.13328	0.82871	H188	0.39313	0.04953	0.33288
C79	0.42163	0.12813	0.81447	H189	0.70330	0.84825	1.67132
C80	0.38971	0.15288	0.77076	H190	0.35610	0.74439	1.10411
C81	0.35893	0.14056	0.57280	H191	0.82839	0.73029	1.61934
C82	0.36001	0.10238	0.42364	H192	0.83252	0.79989	1.44480
C83	0.39232	0.07786	0.46235	H193	0.67140	0.75565	1.74388
N84	0.32755	0.16800	0.52935	H194	0.66685	0.68648	1.92681
C85	0.70214	0.82138	1.52198	H195	0.75457	0.86598	1.29760
C86	0.33344	0.75981	1.23618	H196	0.79378	0.63349	1.89677
C87	0.73575	0.79449	1.53207	H197	0.69638	0.62541	2.02984
C88	0.73252	0.75362	1.63315	H198	0.86467	0.24340	0.71644
C89	0.76583	0.72878	1.64631	H199	0.71150	0.16879	0.98607
C90	0.80186	0.74655	1.58573	H200	0.77375	0.14710	1.19049

C91	0.80472	0.78665	1.49265	H201	0.78809	0.29764	0.39853
C92	0.77237	0.81052	1.45962	H202	0.72621	0.31960	0.19768
C93	0.69710	0.73748	1.73616	H203	0.63521	0.22898	0.69715
C94	0.69429	0.69784	1.84368	H204	0.86115	0.20086	1.13406
C95	0.72639	0.67280	1.84391	H205	0.66099	0.30768	0.13603
C96	0.76218	0.68746	1.73646	H206	0.30102	0.14275	0.11686
O97	0.77838	0.85004	1.35347	H207	0.18032	0.26675	0.23757
C98	0.79437	0.65831	1.72269	H208	0.17790	0.20067	-0.00859
O99	0.72303	0.63293	1.94996	H209	0.33410	0.23720	0.51039
N100	0.82141	0.66148	1.49972	H210	0.33550	0.30305	0.76405
C101	0.83758	0.25780	0.77338	H211	0.21111	0.35823	0.45943
C102	0.80257	0.23297	0.80598	H212	0.20791	0.14067	-0.13348
C103	0.76661	0.24530	0.66848	H213	0.25669	0.36936	0.86444
C104	0.73303	0.22188	0.72782	H214	0.12897	0.73111	1.28650
C105	0.73626	0.18667	0.92004	H215	0.28030	0.80196	0.94939
C106	0.77166	0.17409	1.04121	H216	0.21651	0.82346	0.79284
C107	0.80458	0.19667	0.98987	H217	0.21055	0.67581	1.63511
C108	0.76328	0.28018	0.47027	H218	0.27486	0.65373	1.77527
C109	0.72782	0.29279	0.34965	H219	0.15145	0.81012	0.76351
C110	0.69470	0.27066	0.41478	H220	0.36206	0.70159	1.54565

Table S3. Powder XRD (PXRD) data and BET parameters of the synthesized PyTA-2,3-NA(OH)$_2$ and PyTA-2,6-NA(OH)$_2$ HO-COFs.

HO-COF	S_{BET} (m^2 g^{-1})	d_{110} (nm)	Pore size (nm)	Interlayer distance (Å)
PyTA-2,3-NA(OH)$_2$	480	2.36	2.05	3.73
PyTA-2,6-NA(OH)$_2$	424	2.40	1.81	3.66

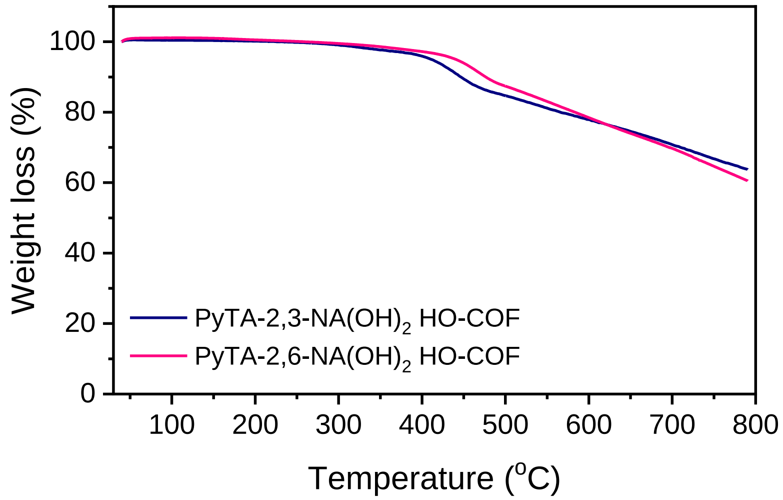

Figure S9. Thermogravimetric (TG) analysis of pyrene-based HO-COFs.

Table S4. Values of $T_{d10\%}$, and char yield of PyTA-2,3-NA(OH)$_2$ and PyTA-2,6-NA(OH)$_2$ HO-COFs.

HO-COFs	$T_{d\,10\%}$ (°C)	Char yield (wt%)
PyTA-2,3-NA(OH)$_2$	446	63.73
PyTA-2,6-NA(OH)$_2$	476	60.46

5. Quartz Crystal Microbalance (QCM) Gas Sensor Experimental Setup

The QCM sensor is a mass sensing technology that operates at room temperature and offers fast response time, sensitivity, and a low detection limit in the nanogram range. Furthermore, the QCM integrated with materials nanoarchitectonics to fabricate a sensor system, enhancing the sensitivity and selectivity of detecting a particular/individual analyte in a complex gas mixture. [S1-S3]. The QCM sensor uses the piezoelectric effect to measure the change in mass of the deposited nanostructured material layer by measuring the change in resonance frequency of the quartz crystal, and the Sauerbrey equation is utilized to determine the relationship between mass and resonant frequency. QCM sensor technology can precisely measure the change in oscillation frequency induced by the additional adsorbed mass of the chemical-vapor analyte at the nanogram range. Sauerbrey studied the relationship between ΔF (Hz) and the mass per unit area (Δm, g cm^{-2}) deposited onto the Au electrode of the QCM sensor at a fundamental resonant frequency, F_0, as described in Eq. (S1) [S4].

$$\Delta F = -\frac{2NF_0^2}{\sqrt{\rho\mu}}\frac{\Delta m}{A} \dots\dots\dots\dots\dots\dots (S1)$$

$$\Delta F = F_1 - F_0, \dots\dots\dots\dots\dots\dots.\,.\dots (S2)$$

where, N, F_0, ρ, μ, and A express the harmonic overtone, the fundamental resonance frequency of the crystal (Hz), the density of quartz (2.649 g cm^{-3}), elastic shear modulus (2.947 × 10^{11} g cm^{-1} s^{-2}), and electrode surface area (5 mm diameter, 0.196 cm^2), respectively. To determine the mass of the sample deposited on the QCM electrode, ΔF was recorded after drop-coating of the PyTA-2,3-NA$(OH)_2$ and PyTA-2,6-NA$(OH)_2$ HO-COFs samples as 10,036.5 and 11,466.7 Hz; thus the mass of the PyTA-2,3-NA$(OH)_2$ and PyTA-2,6-NA$(OH)_2$ HO-COFs deposited on the Au surface of the electrode was 3.18 and 3.64 µg, respectively. All the recorded frequencies were normalized by mass to determine sensor sensitivity and selectivity.

The frequency change caused as a result of the additional adsorbed mass was measured using the QCM sensor technology (AT-cut 30 MHz, model QCM922A, SEIKO EG&G Co. Ltd., Japan) to detect ethylenediamine (EDA) vapor. Prior to applying the coating to the COF samples, the uncoated Au electrodes of the QCM sensor were soaked in a 3:1 ethanol/water mixture and sonicated for 30 minutes, then vacuum-dried at 60 °C in an oven. After recording their fundamental frequencies (F_0), the masses of the COF samples coated on the surface of the Au electrode were determined using the Sauerbrey equation (Eq. (1)) based on the values of F_0. Aqueous solutions of Nafion binder (1 mL/0.05 wt%) were mixed with homogeneous suspension solutions of COF samples (2 mg) to coat the electrodes,

and the mixture was sonicated for 30 min. The COF samples (5 μL) were drop-coated onto the uncoated Au electrodes at room temperature to fabricate QCM electrodes, which were then left undisturbed for 30 min. Subsequently, the electrodes were vacuum-dried overnight at 60 °C in a convection oven. To perform QCM sensor tests, electrodes modified with PyTA-2,3-NA$(OH)_2$ or PyTA-2,6-NA$(OH)_2$ HO-COFs were connected inside the testing vessel (Figure S10). The frequencies of these electrodes after coating were then measured as F_1 under a flow of N_2 gas. A steady baseline (± 1 Hz min^{-1}) was observed, suggesting that the COFs were being adhered to the Au surface of the QCM electrodes. All measurements were conducted under ambient conditions. The glass vessel's temperature and relative humidity were recorded at 24 ± 2 °C and 52 ± 4%, respectively.

By measuring the frequency change (ΔF, Hz) of the QCM caused by vapor additional mass at room temperature, the gas-sensing properties of PyTA-2,3-NA$(OH)_2$ and PyTA-2,6-NA$(OH)_2$ HO-COFs towards EDA vapors were studied in the presence of other vaporized substances including acetonitrile, ammonia, benzene, chloroform, ethanol, formaldehyde, formic acid, methanol, pyridine, toluene, water. To desorb the chemical vapor analyte molecules and achieve a baseline frequency response, a flow of N_2 gas was purged through the testing glass vessel and applied to the vapor analyte-saturated PyTA-2,3-NA$(OH)_2$ or PyTA-2,6-NA$(OH)_2$-modified QCM electrodes. The return of the electrode to its initial frequency was taken as an indication of complete desorption of the chemical-vapor analyte. During sequential injection of the liquid analyte, the time-dependent frequency was automatically recorded on a PC running the WinQCM software measurement program.

The gaseous chemical analytes were obtained at ambient temperature (24 ± 2 °C) by injecting an appropriate volume of the liquid analyte using a Hamilton microliter syringe (Hamilton Company Inc., Switzerland). The vapor concentration was calculated in ppm based on its density and mass concentration according to the following formula (Eq. (S3)) [S5].

$$C_{ppm} = \frac{22.4\rho T V_s}{273MV} \times 10^3 \dots\dots\dots\dots\dots\dots. (S3)$$

where, C_{ppm} refers to the analyte concentration (ppm), ρ is the density of injected liquid analyte (g mL^{-1}), T is the working temperature (K), M is the molecular weight of injected chemical-analyte (g), and Vs and V represent the volumes of the injected chemical-analyte (μL) and the working glass vessel volume (L), respectively.

The structural features of HO-COF materials can influence the adsorption rate of EDA vapor, which is controlled by surface area, porosity, and morphology. As a result, EDA adsorption can be

considered a pseudo-first-order mass transfer between the vapor phase and the HO-COF material, which can be investigated in real-time by measuring the frequency immediately after injecting EDA into the glass vessel. A pseudo-first-order kinetic model was used to fit the experimental ΔFs of the QCM sensor. Equations S4-6 were used to calculate the pseudo-first-order kinetic rate constant (k_1), which represents the initial rate of uptake of EDA vapors ($\Delta F_t/\Delta F_\infty$) [S5].

$$\frac{\Delta F_t}{\Delta F_\infty} = 1 - e^{-k_1 t} \dots\dots\dots\dots\dots\dots\dots\dots (S4)$$

$$\ln(1 - \frac{\Delta F_t}{\Delta F_\infty}) = -k_1 t \dots\dots\dots\dots\dots\dots (S5)$$

$$\Delta F_t = F - F_t \text{ and } \Delta F_\infty = F - F_\infty \dots (S6)$$

where, ΔF_t and ΔF_∞ refer to the frequency changes for vapor uptake at time (t) and equilibrium, respectively. k_1 (min^{-1}) is the adsorption rate constant of the pseudo-first-order kinetic model. F is the oscillating crystal frequency before exposure to the analyte at t = 0, and F_t and F_∞ are the crystal frequency after injection of the analyte at a time (t) and equilibrium, respectively.

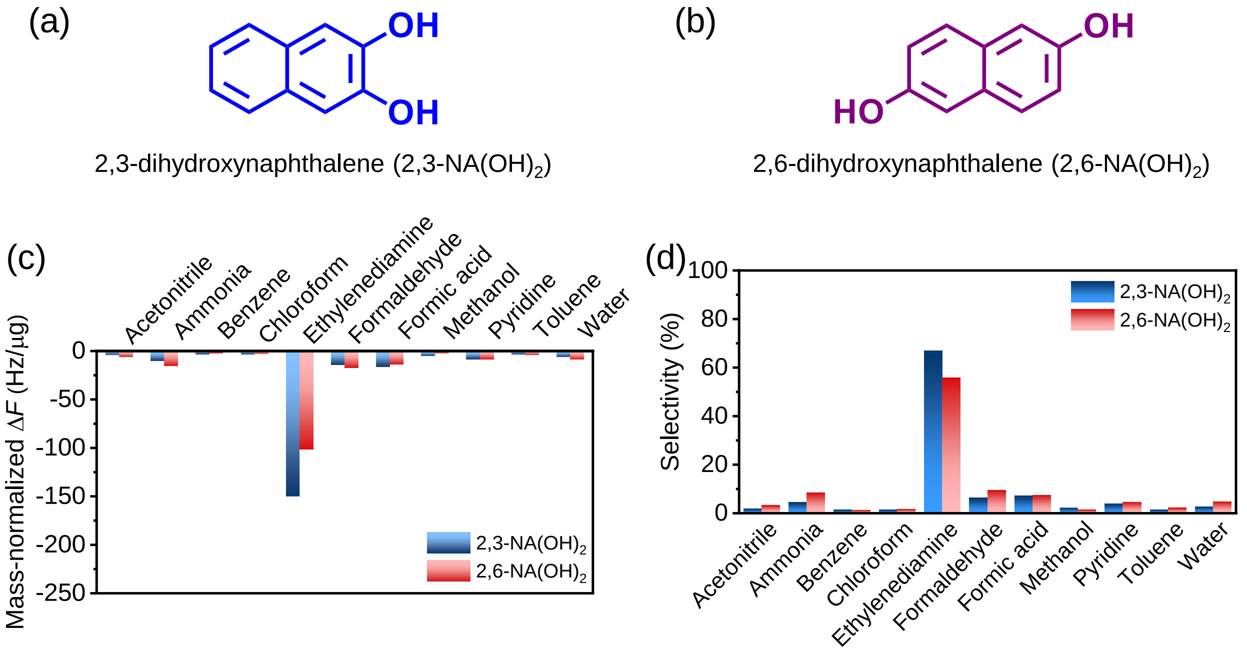

Figure S10. (a, b) The chemical structure of the model compounds 2,3-dihydroxynaphthalene (2,3-NA(OH)$_2$) and 2,6-dihydroxynaphthalene (2,6-NA(OH)$_2$), respectively. (c) Mass-normalized Δ*F*s and (d) selectivity test of 2,3-NA(OH)$_2$ and 2,6-NA(OH)$_2$-based QCM sensors towards chemical-vapor analytes at room temperature. The frequency was recorded after the injection of chemical-vapor analytes at 100 ppm.

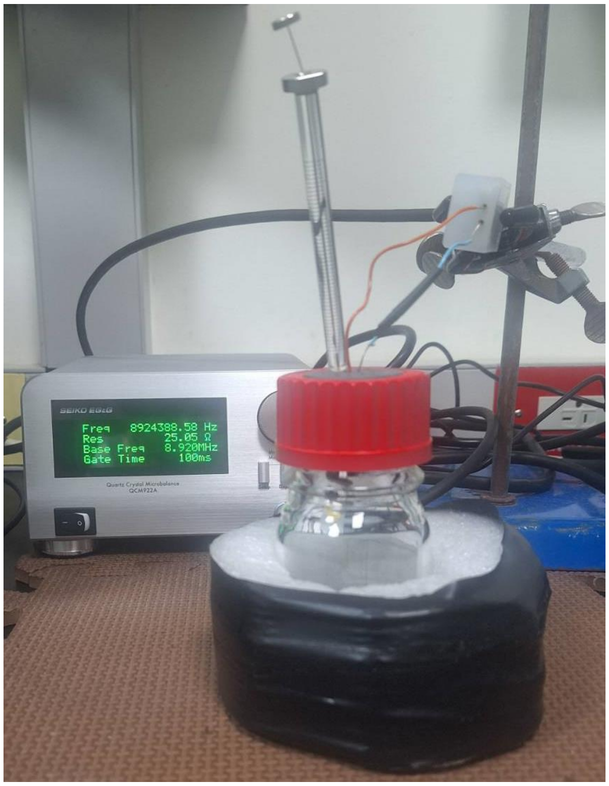

Figure S11. The PyTA-2,6-NA(OH)$_2$ and PyTA-2,3-NA(OH)$_2$ HO-COF-modified QCM sensor experimental setup.

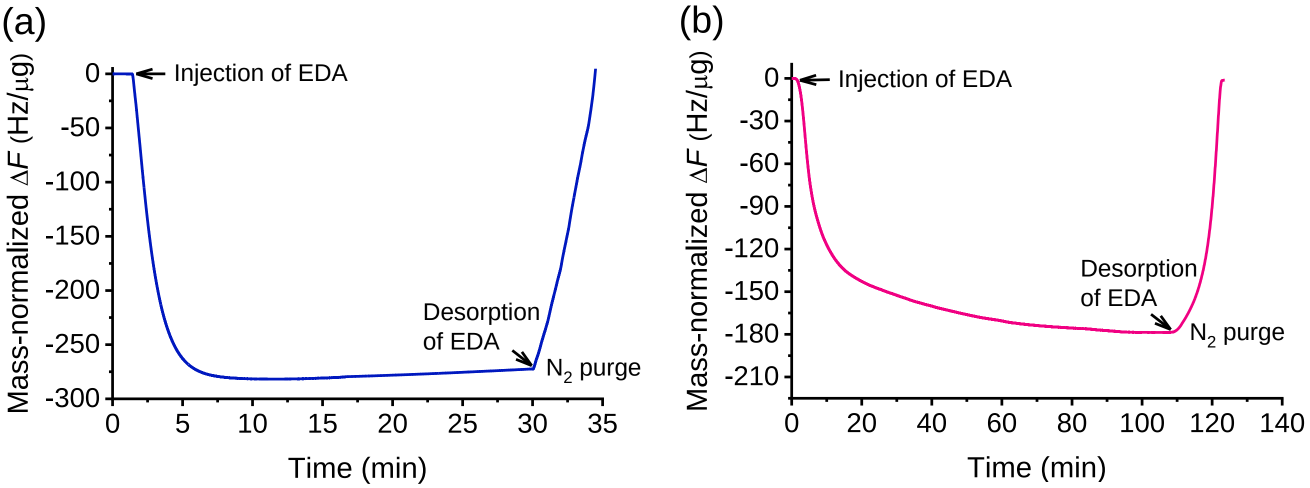

Figure S12. Response and recovery curves of the QCM sensor upon exposure to 100 ppm of the EDA using (a) PyTA-2,3-NA(OH)$_2$ and (b) PyTA-2,6-NA(OH)$_2$ HO-COFs at ambient temperature and pressure. The recovery was achieved by purging the system with high-purity N_2 gas.

Table S5. Summary of hazardous EDA vapor sensing performance for various nanostructured materials reported in the representative literature.

Material	Sensing technique	EDA concentration	LOD	Ref.
PyTA-2,3-NA(OH)$_2$	QCM sensor	100 ppm	2.9 ppm	This work
OPTA-MSA loaded PVA hydrogel*	--	160 ppm	3.2 ppm	S6
Tetrakis-carboxyphenyl porphyrin (TCPP)	Optical waveguide sensor	1000 ppm	0.1 ppm	S7
Perylenediimide-camphorsulfonic acid complex	Conductometric sensor	100 ppm	0.86 ppm	S8
Tb(acacn)@ZY*	Luminescence sensor	5 mL	2% (volume ratio)	S9
MoO$_3$/rGO*	Conductometric sensor	100 ppm	0.235 ppm	S10
Zn(salen)-type complex	Optical chemosensors	5000 ppm	6.6 ppm	S11
THPP-BCP/TiO$_2$*	Optical waveguide sensor	1 ppm	1 ppb	S12
5,10,15,20-tetrakis-(4-methoxyphenyl) porphyrin	Optical waveguide sensor	1000 ppm	1 ppm	S13
1-Naphthylisothiourea derivative	High-performance liquid chromatography	50.85 ppm	--	S14
Nitrated polythiophene (NPTh)	UV-vis absorption spectroscopy	15,900 ppm	5.6 ppm	S15

* OPTA-MSA loaded PVA hydrogel: *O*-phthalaldehyde-mercaptosuccinic acid loaded cross-linked polyvinyl alcohol hydrogel

* Tb(acacn)@ZY: Terbium-acetylacetonate complexes within the cavities of Y-type zeolite

* MoO$_3$/rGO: MoO$_3$ nanoribbon modified with rGO nanosheets

* THPP-BCP/TiO$_2$: Hydroxyphenyl porphyrin (THPP)-bromocresol purple (BCP)/TiO$_2$ gel composite film

* H$_2$btm: Di(1*H*-tetrazol-5-yl)methane

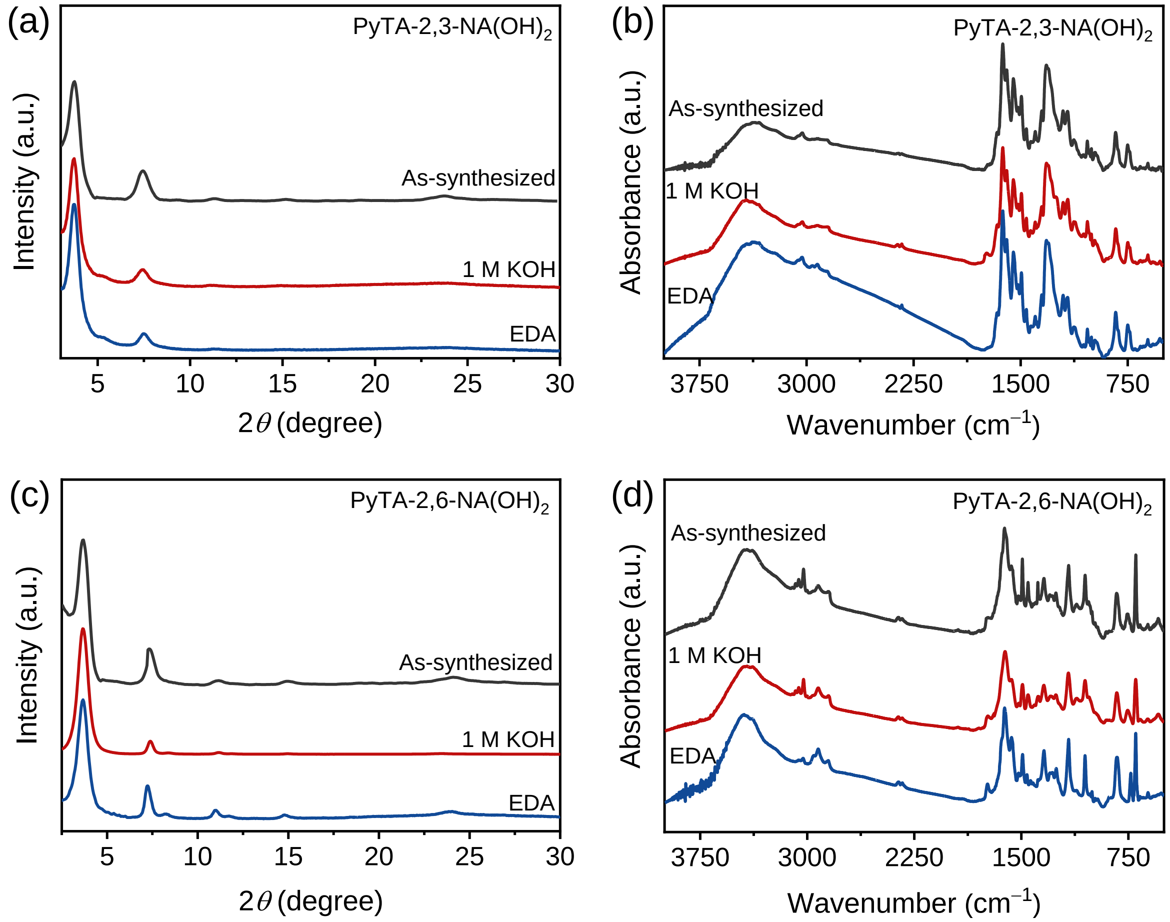

Figure S13. PXRD and FTIR measurements of (a, b) PyTA-2,3-NA$(OH)_2$ and (c, d) PyTA-2,6-NA$(OH)_2$ HO-COFs before and after immersing in aqueous solutions of KOH and EDA for 24h.

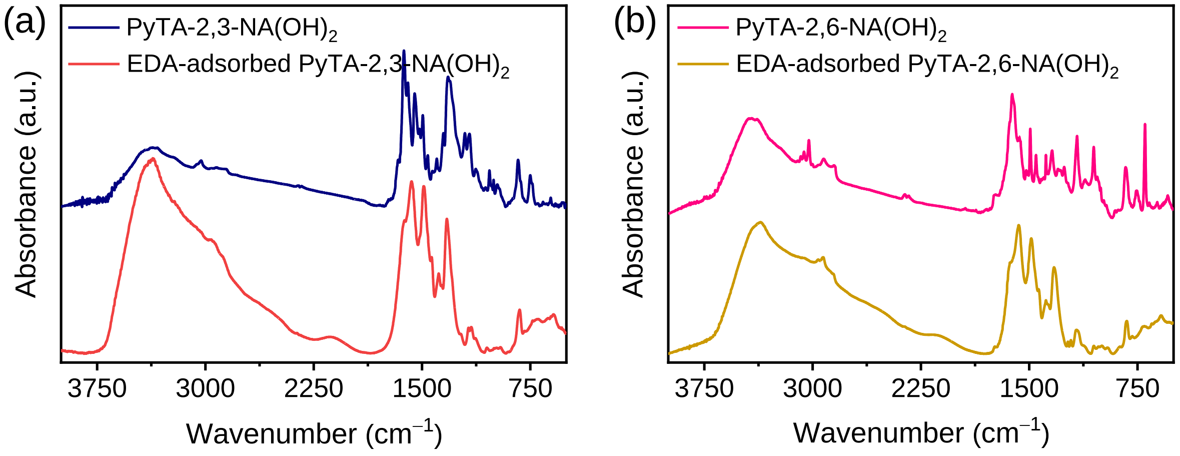

Figure S14. FTIR spectra of (a, b) the PyTA-2,3-NA$(OH)_2$ and PyTA-2,6-NA$(OH)_2$ HO-COFs before and after exposure to EDA vapor measured at room temperature.

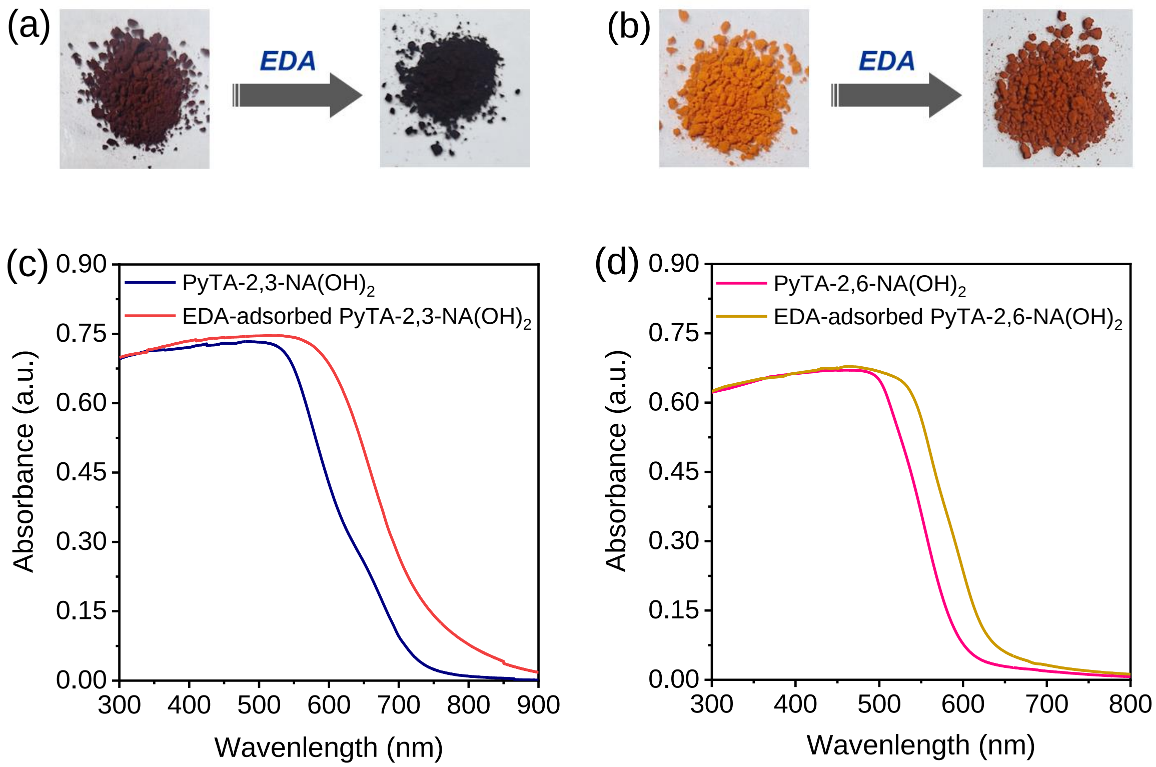

Figure S15. Photographs of the (a) dry PyTA-2,3-NA$(OH)_2$ HO-COF and (b) dry PyTA-2,6-NA$(OH)_2$ HO-COF before and after EDA exposure measured at room temperature, respectively. UV-vis diffuse reflectance spectroscopy plots of the (c) dry PyTA-2,3-NA$(OH)_2$ HO-COF and (d) dry PyTA-2,6-NA$(OH)_2$ HO-COF before and after EDA vapor exposure measured at room temperature.

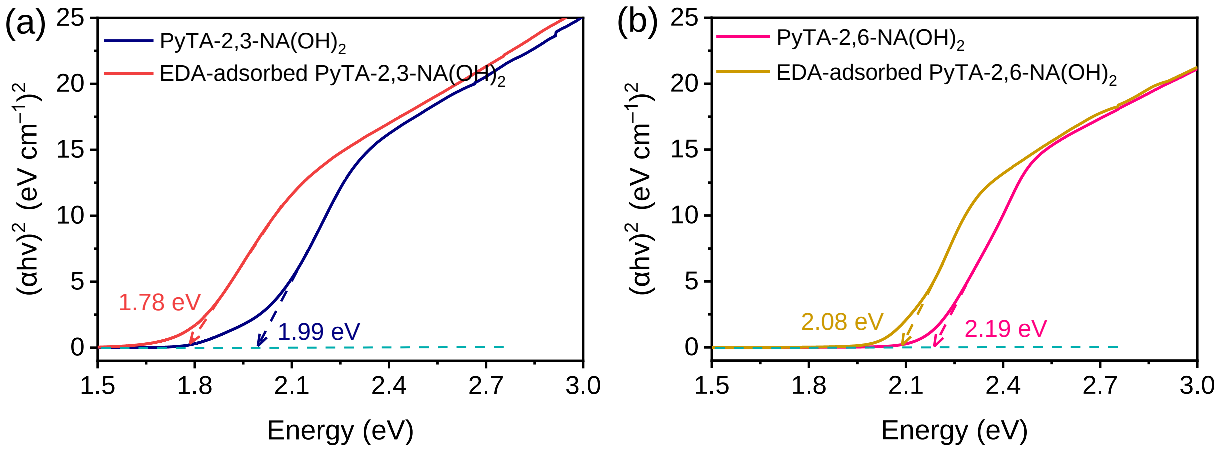

Figure S16. Tauc plots from the UV-vis diffuse reflectance spectroscopy plots of the (a) dry PyTA-2,3-NA$(OH)_2$ and (b) dry PyTA-2,6-NA$(OH)_2$ HO-COF before and after EDA vapor exposure measured at room temperature.

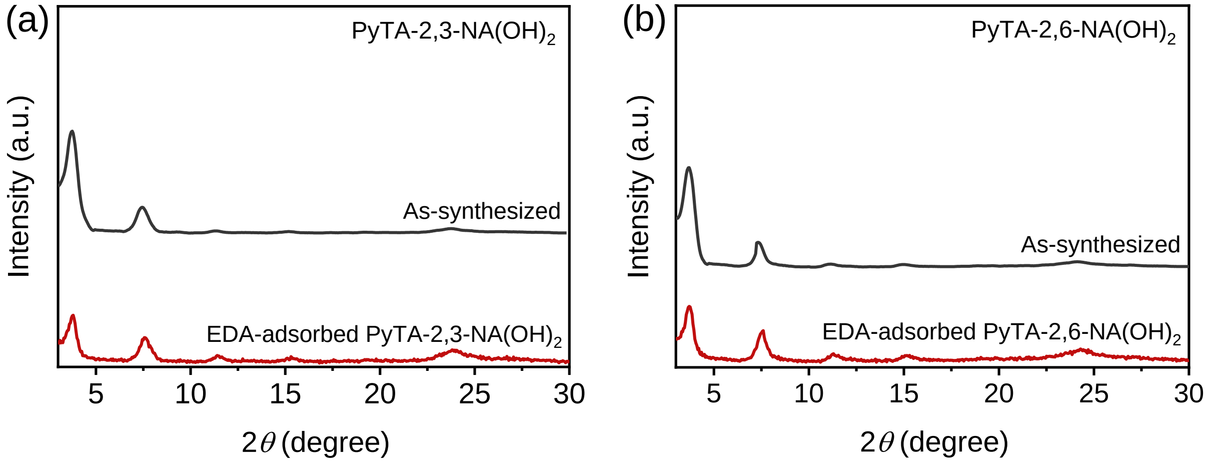

Figure S17. PXRD patterns of the (a) dry PyTA-2,3-NA$(OH)_2$ HO-COF and EDA-adsorbed PyTA-2,3-NA$(OH)_2$, and (b) dry PyTA-2,6-NA$(OH)_2$ HO-COF and EDA-adsorbed PyTA-2,6-NA$(OH)_2$ measured at room temperature.

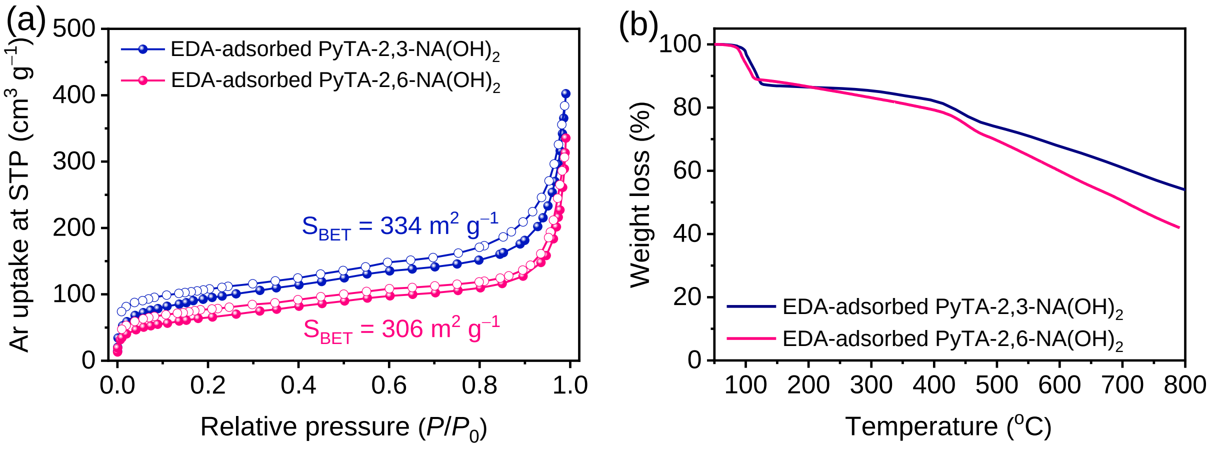

Figure S18. (a) Ar adsorption-desorption isotherms of the EDA-adsorbed PyTA-2,3-NA$(OH)_2$ and EDA-adsorbed PyTA-2,6-NA$(OH)_2$ HO-COF. (b) TGA curves of the PyTA-2,3-NA$(OH)_2$ and PyTA-2,6-NA$(OH)_2$ HO-COFs before and after exposure to EDA vapor.

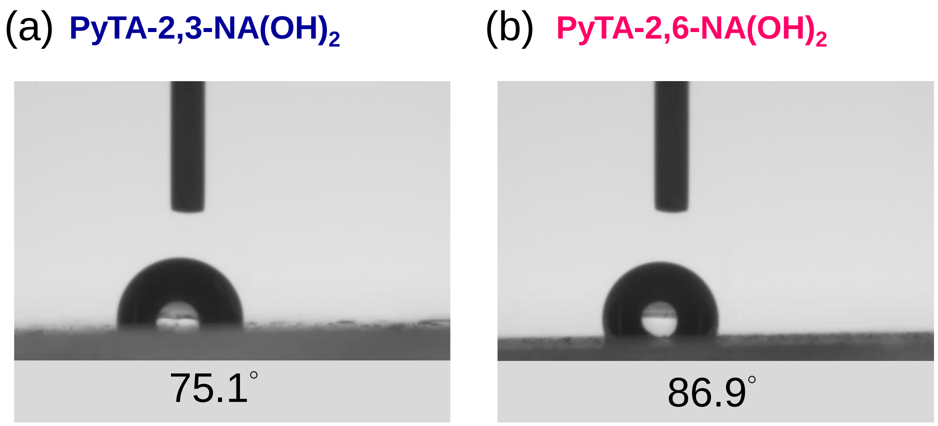

Figure S19. Water contact angle measurement of (a) PyTA-2,3-NA$(OH)_2$ and (b) PyTA-2,6-NA$(OH)_2$ HO-COFs.

6. Computational Analysis Setup. Density functional theory (DFT) calculations were performed using the Vienna ab initio simulation package (VASP) to determine the most stable possible adsorption energy of 11 organic molecules into PyTA-2,3-NA$(OH)_2$ and PyTA-2,6-NA$(OH)_2$ HO-COF structures [S16]. The generalized gradient approximation (GGA) in the formalism of Perdew-Burke-Ernzerhof (PBE) was adopted to describe the electronic exchange-correlation energy. The projector-augmented wave (PAW) pseudo-potentials were chosen to describe ionic cores [S17, S18]. We have utilized the standard PBE functional method to improve the van der Waals interactions correctly by Becke-Jonson damping potential with the DFT-D3 method [S19]. The convergence criterion for the self-consistent iteration was 10^{-5} eV. The calculations were carried out with a plane-wave basis set cutoff energy set to 400 eV and with the Gaussian smearing method of 0.05 eV width in order to assure well-converged total energy and force values.

The structural optimization of the HO-COF bulk unit cell of PyTA-2,6-NA$(OH)_2$ and PyTA-2,3-NA$(OH)_2$ has a two-layered crystal structure and was performed with the Brillion Zone sampled using a 1×1×3 Monkhorst-Pack k-point grid. The predicted lattice parameters (a,b,c) of PyTA-2,6-NA$(OH)_2$ are a = 36.162 Å, b = 30.970 Å, c = 6.969 Å, and PyTA-2,3-NA$(OH)_2$ are a = 35.701 Å, b = 30.482 Å, c = 6.999 Å. The distance between the two layers in the unit cell model of both PyTA-2,6-NA$(OH)_2$ and PyTA-2,3-NA$(OH)_2$ is ~ 3.5 Å, and their optimized structures are shown in Figure 1. Both models have two distinct hydroxyl adsorption sites to bind the organic molecules. The adsorption site of the OH-functional group in anthracene at PyTA-2,6-NA$(OH)_2$ is one, and the other is 2 OH groups side by side at PyTA-2,3-NA$(OH)_2$. The adsorption energy of the molecule (E_{ads}) was determined from Eq. (S7).

$$E_{ads} = (E_{COF} + E_{molecule}) - E_{(COF+molecule)} \dots\dots\dots\dots\dots (S7)$$

where, E_{COF} represents the energy of the PyTA-2,6-NA$(OH)_2$ unit cell, $E_{molecule}$ is the energy of the adsorbate in the gas phase, and $E_{(COF+molecule)}$ is the total energy of the adsorbate bound with the OH group in PyTA-2,6-NA$(OH)_2$.

Table S6. Binding energetics parameters for the most stable configuration of the complex EDA-adsorbed PyTA-2,3-NA$(OH)_2$, and EDA-adsorbed PyTA-2,6-NA$(OH)_2$ and its isolated components: EDA and HO-COFs. Energies are in eV, and bond distances (*d*) are in Ångstroms (Å).

	Ethylenediamine	PyTA-2,3-NA$(OH)_2$	PyTA-2,6-NA$(OH)_2$
Acetonitrile	--	-0.37	-0.31
Ammonia	--	-0.65	-0.54
Benzene	--	-0.41	-0.25
CCl_4	--	-0.35	-0.32
Ethylenediamine	--	-1.39	-1.01
Formaldehyde	--	-0.59	-0.49
Formic acid	--	-0.78	-0.63
Methanol	--	-0.53	-0.43
Pyridine	--	-0.60	-0.55
Toluene	--	-0.33	-0.26
Water	--	-0.57	-0.34
$d_{C-O1}{}^{Anthracene}$	--	1.35	1.38
$d_{O1-H}{}^{hydroxy}$	--	1.0	1.1
$d_{C-O2}{}^{Anthracene}$	--	1.38	1.37
$d_{O2-H}{}^{hydroxy}$	--	1.1	1.05
$d_{C-C}{}^{EDA}$	1.53	1.53	1.53
$d_{C-N1}{}^{EDA}$	1.47	1.47	1.47
$d_{C-N2}{}^{EDA}$	1.48	1.48	1.47
$d_{N1-OH1}{}^{EDA@HO-COF}$	--	1.86	--
$d_{N2-OH2}{}^{EDA@HO-COF}$	--	1.5	1.6
E_{ads}	--	-32.1	-23.4

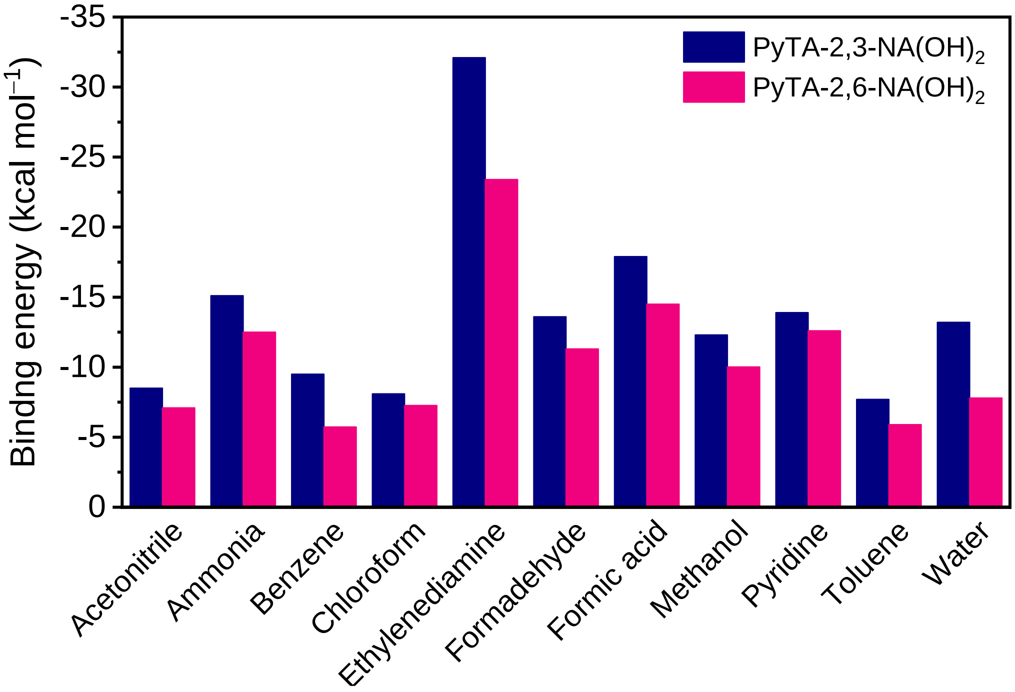

Figure S20. The most stable possible adsorption energy of eleven chemical-vapor analytes into the nanofibers of PyTA-2,6-NA$(OH)_2$ and PyTA-2,3-NA$(OH)_2$ HO-COFs.

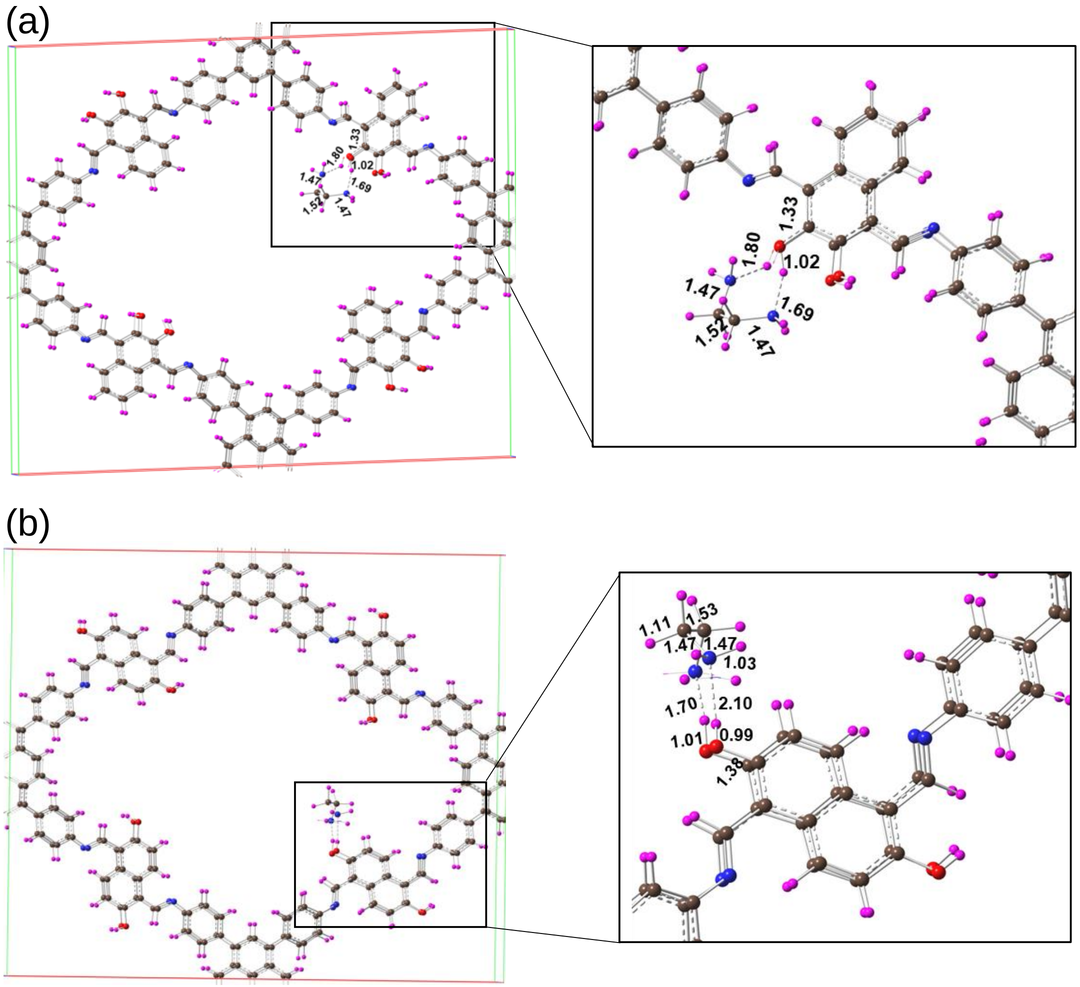

Figure S21. (a, b) EDA interactions with the neighboring OH groups in adjacent layers of PyTA-2,3-NA$(OH)_2$ and PyTA-2,6-NA$(OH)_2$ HO-COFs, respectively. Dimensions are given in nanometers, and bond length in Ångstroms (Å).

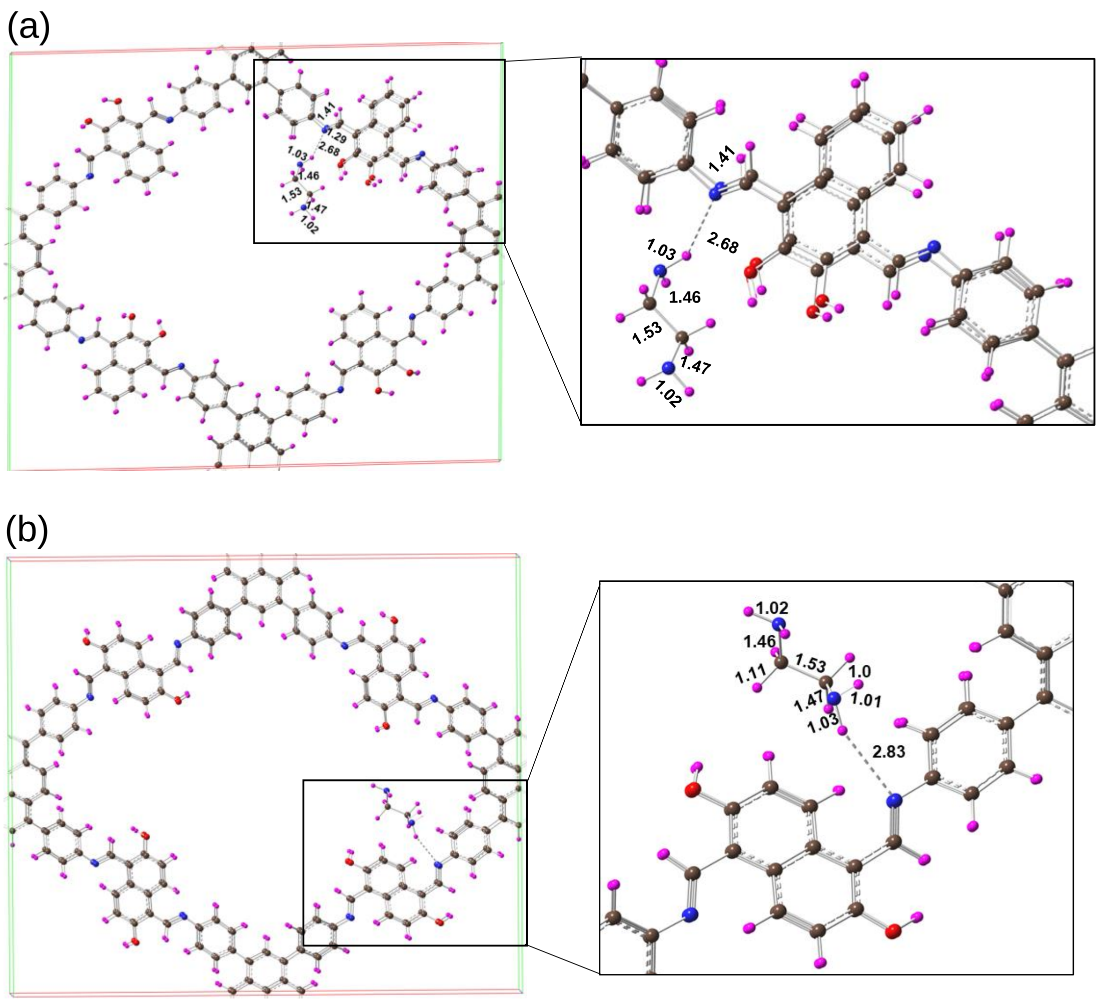

Figure S22. (a, b) Binding of the EDA molecule with the imine N groups of PyTA-2,3-NA$(OH)_2$ and PyTA-2,6-NA$(OH)_2$, respectively. Dimensions are given in nanometers, and bond length in Ångstroms (Å).

SI References

(S1) Torad, N. L.; Zhang, S.; Amer, W. A.; Ayad, M. M.; Kim, M.; Kim, J.; Ding, B.; Zhang, X.; Kimura, T.; Yamauchi, Y. Advanced nanoporous material-based QCM devices: A new horizon of interfacial mass sensing technology. Advanced Materials Interfaces 2019, 6 (20), 1900849. DOI: https://doi.org/10.1002/admi.201900849.

(S2) Kotp, M. G.; Torad, N. L.; Lüder, J.; El-Amir, A. A. M.; Chaikittisilp, W.; Yamauchi, Y.; El-Mahdy, A. F. M. A phenazine-conjugated microporous polymer-based quartz crystal microbalance for sensitive detection of formaldehyde vapors at room temperature: an experiment and density functional theory study. Journal of Materials Chemistry A 2023, 11 (2), 764–774, 10.1039/D2TA07966F. DOI: https://doi.org/10.1039/D2TA07966F.

(S3) Torad, N. L.; Ding, B.; El-Said, W. A.; El-Hady, D. A.; Alshitari, W.; Na, J.; Yamauchi, Y.; Zhang, X. MOF-derived hybrid nanoarchitectured carbons for gas discrimination of volatile aromatic hydrocarbons. Carbon 2020, 168, 55–64. DOI: https://doi.org/10.1016/j.carbon.2020.05.013.

(S4) Chowdhury, S.; Torad, N. L.; Godara, M.; El-Amir, A. A. M.; Gumilar, G.; Ashok, A.; Rezaul Karim, M.; Abdullah Alnaser, I.; Chaikittisilp, W.; Ray, N.; et al. Hierarchical bimetallic metal-organic frameworks with controllable assembling sub-units and interior architectures for enhanced ammonia detection. Chemical Engineering Journal 2024, 480, 147990. DOI: https://doi.org/10.1016/j.cej.2023.147990.

(S5) Torad, N. L.; El-Hosainy, H.; Esmat, M.; El-Kelany, K. E.; Tahawy, R.; Na, J.; Ide, Y.; Fukata, N.; Chaikittisilp, W.; Hill, J. P.; et al. Phenyl-modified carbon nitride quantum nanoflakes for ultra-highly selective sensing of formic acid: A combined experimental by QCM and density functional theory study. ACS Applied Materials & Interfaces 2021, 13 (41), 48595–48610. DOI: 10.1021/acsami.1c12196.

(S6) Ke, Y.; Liu, Y.; Zu, B.; Lei, D.; Wang, G.; Li, J.; Ren, W.; Dou, X. Electronic tuning in reaction-based fluorescent sensing for instantaneous and ultrasensitive visualization of ethylenediamine. Angewandte Chemie International Edition 2022, 61 (29), e202203358. DOI: https://doi.org/10.1002/anie.202203358.

(S7) Ma, Q.; Zhang, Y.; Abudukeremu, H.; Maimaiti, A.; Wumaier, K.; Nizamidin, P.; Yimit, A. Detection of ethylenediamine vapor by optical waveguide sensor based on tetrakis-carboxylphenyl porphyrin film. Journal of Applied Spectroscopy 2020, 87 (5), 986–993. DOI: https://doi.org/10.1007/s10812-020-01098-9.

(S8) Huang, Y.; Liu, X.; Wang, Q.; Fu, J.; Zhao, L.; Liu, Z.; Jin, D. Highly responsive ethylenediamine vapor sensor based on a perylenediimide–camphorsulfonic acid complex via ionic self-assembly. Journal of Materials Chemistry C 2017, 5 (30), 7644–7651. DOI: https://doi.org/10.1039/C7TC02580G.

(S9) Li, P.; Yang, D.; Li, H. Luminescence ethylenediamine sensor based on terbium complexes entrapment. Dyes and Pigments 2016, 132, 306–309. DOI: https://doi.org/10.1016/j.dyepig.2016.05.013.

(S10) Liu, H.; Liu, J.; Liu, Q.; Li, Y.; Zhang, G.; He, C. Conductometric gas sensor based on MoO3 nanoribbon modified with rGO nanosheets for ethylenediamine detection at room temperature. Nanomaterials 2023, 13 (15), 2220. https://doi.org/10.3390/nano13152220.

(S11) Attinà, A.; Oliveri, I. P.; Gaeta, M.; Di Bella, S. Sensitive and discriminative fluorescent detection of volatile primary aliphatic diamine vapors from monoamines. Molecules 2024, 29 (24), 5947. https://doi.org/10.3390/molecules29245947.

(S12) Kutilike, B.; Yiming, K.; Tuerdi, G.; Abdurahman, R.; Nizamidin, P.; Yimit, A. A novel TiO_2-modified THPP-BCP composite optical waveguide sensor for the determination of ethylenediamine at ppb level. Analytical Sciences 2024, 40 (2), 291–300. DOI: https://doi.org/10.1007/s44211-023-00458-7.

(S13) Mamtmin, G.; Kari, N.; Abdurahman, R.; Nizamidin, P.; Yimit, A. 5, 10, 15, 20-tetrakis-(4-methoxyphenyl) porphyrin film/K^+ ion-exchanged optical waveguide gas sensor. Optics & Laser Technology 2020, 128, 106260. DOI: https://doi.org/10.1016/j.optlastec.2020.106260.

(S14) Andersson, K.; Hallgren, C.; Levin, J.-O.; Nilsson, C.-A. Determination of ethylenediamine in air using reagent-coated adsorbent tubes and high-performance liquid chromatography on the 1-naphthylisothiourea derivative. American Industrial Hygiene Association Journal 1985, 46 (4), 225–229. DOI: https://doi.org/10.1080/15298668591394707.

(S15) Jin, Y.-J.; Kwak, G. Detection of biogenic amines using a nitrated conjugated polymer. Sensors and Actuators B: Chemical 2018, 271, 183–188. DOI: 10.1016/j.snb.2018.05.091.

(S16) Kresse, G.; Furthmüller, J. Efficient iterative schemes for ab initio total-energy calculations using a plane-wave basis set. Physical Review B 1996, 54 (16), 11169–11186. DOI: https://doi.org/10.1103/PhysRevB.54.11169.

(S17) Lee, C.; Yang, W.; Parr, R. G. Development of the Colle-Salvetti correlation-energy formula into a functional of the electron density. Physical Review B 1988, 37 (2), 785–789. DOI: https://doi.org/10.1103/PhysRevB.37.785.

(S18) Perdew, J. P.; Burke, K.; Ernzerhof, M. Generalized gradient approximation made simple. Physical Review Letters 1996, 77 (18), 3865–3868. DOI: https://doi.org/10.1103/PhysRevLett.77.3865.

(S19) Tkatchenko, A.; DiStasio, R. A.; Car, R.; Scheffler, M. Accurate and efficient method for many-body van der Waals interactions. Physical Review Letters 2012, 108 (23), 236402. DOI: https://doi.org/10.1103/PhysRevLett.108.236402.
